# Magneto-hydrothermal triple-convection in a W-shaped porous cavity containing oxytactic bacteria

**DOI:** 10.1038/s41598-022-18401-7

**Published:** 2022-10-27

**Authors:** Nirmalendu Biswas, Dipak Kumar Mandal, Nirmal K. Manna, Ali Cemal Benim

**Affiliations:** 1 Department of Power Engineering, Jadavpur University, Salt Lake, Kolkata, 700106 India; 2 Department of Mechanical Engineering, College of Engineering and Management, Kolaghat, 721171 India; 3 Department of Mechanical Engineering, Jadavpur University, Kolkata, 700032 India; 4 Department of Mechanical and Process Engineering, Duesseldorf University of Applied Sciences, 40476 Duesseldorf, Germany

**Keywords:** Engineering, Mathematics and computing

## Abstract

Bioconvective heat and mass transport phenomena have recently been the subject of interest in diverse fields of applications pertaining to the motion of fluids and their thermophysical properties. The transport processes in a system involving triple convective phenomena, irregular geometry, and boundary conditions constitute a complex phenomenon. This work aims to explore the mixed thermo-bioconvection of magnetically susceptible fluid containing copper nanoparticles and oxytactic bacteria in a novel W-shaped porous cavity. The buoyant convention is generated due to the isothermal heating at the wavy bottom wall, whereas the mixed convection is induced due to the shearing motion of the top-cooled sliding wall. Furthermore, the bioconvection is induced due to the manifestation of oxytactic bacteria or organisms. The inclined sidewalls are insulated. The geometry is packed with water based Cu nanoparticle mixed porous structure, which is subjected to a magnetizing field acted horizontally. The complex transport equations are transformed into nondimensional forms, which are then computed using the finite volume-based developed code. The coupled triple-convective flow physics are explored for a wide range of involved controlling parameters, which could provide helpful insight to the system designer for its proper operation. The shape of geometry can be considered one of the important parameters to control the heat and mass transport phenomena. In general, the influence of amplitude (*δ*) is more compared to the waviness number (*m*) of the undulations. The magnitude of heat (Nu) and mass (Sh) transfer rate for the W-shaped cavity is high compared to conventional square and trapezoidal-shaped cavities. The output of the analysis could be very helpful for the designer for modeling devices operating on nanotechnology-based bioconvection, microbial fuel cells, and others.

## Introduction

With technological advancement, bioconvective heat and mass transport phenomena have been the subject of interest in diverse fields of applications pertaining to the motion of fluids and their thermophysical properties. For instance, the applications could be found in the diverse fields of engineering, nanotechnology-based bioconvective processes, food processing systems, chemical reactors, microbial fuel cells, bio-energy systems, enhanced oil recovery, biological wastes processing, microfluidic devices, medical science, and others^1–4^. In general, the term ‘bioconvection’ denotes a macroscopic fluid motion as a result of swimming of the suspended bacteria or motile microorganisms (which are self-propelled in nature). Depending on the type stimulant like light, gravity, oxygen, or chemical attraction, different type of swimming pattern or bioconvective phenomena arises. For the last several years, different types of bioconvection have been studied by various researchers. In general, oxytactic bacteria are propelled in the upwards direction and gathered on the upper surface due to the availability of atmospheric oxygen. Therefore, the growth of their concentration near the upper fluid layer causes the unstable, and it creates the formation of bioconvection^5–11^.

In general, bioconvective phenomena are further influenced by the buoyant (or free) convective flow (due to the existence of thermal gradient)^12^. Avramenko and Kuznetsov^13^ studied the thermo-bioconvective phenomena in a thin fluid film containing oxytactic bacteria and thermal gradient. He found that the bioconvective process is dictated by the thermal as well as bioconvective Rayleigh numbers. When there is an external shear force in the form of wall translation, fluid flow moves under the wall-shearing action, which is dictated by the velocity of the wall translation^14^ and it results in the forced convection. Combining the buoyant and forced convection, the convective process is termed mixed convection^15,16^. Altogether, these three convective processes (i.e. bioconvection, free, and forced convection) are defined as triple-convection. There are limited numbers of studies on the combined convective process due to the complexity of the involved flow physics. The complexity becomes much more in the presence of a flow-hindering medium-like porous substance. Modeling of the porous medium is another important issue for correct capturing of the flow-physics^17,18^. During the last several years, various researchers have studied the bioconvection phenomena in the presence of oxytactic bacteria and fluid-saturated porous substances. On the above background, Kuznetsov et al.^19^ analyzed the oxytactic bioconvective process during the falling plume in a deep space packed with fluid-soaked porous structure and found a decrease in oxygen concentration but a rise in cell density from the boundary towards the center of the plume. On a similar background, Becker et al.^20^ numerically studied the falling oxytactic bioconvective plume through porous layers and observed the highest cell concentrations and lowest oxygen concentrations in the plume center. Oxytactic thermo-bioconvection in a fluid-saturated porous horizontal sheet heated from below has been examined by Kuznetsov^21^ and obtained the correlation inbetween thermal Rayleigh number and bioconvection Rayleigh number. Later, Sheremet and Pop^22^ examined the thermal-bioconvective process in a square cavity jam-packed with porous layers and oxytactic bacteria. They observed that the inclusion of oxytactic bacteria decreases (~ 15%) heat transfer at higher thermal Rayleigh number (Ra) and lower bioconvective Rayleigh number (Rb); whereas a rising tendency in heat transfer (~ 9%) is noted for lower Ra and higher Rb. Furthermore, Balla et al.^23,24^ explored the thermo-bioconvective phenomena in a porous cavity having oxytactic microorganisms under the thermal radiation and found that the density of oxygen, as well as bioconvection strength, is higher with Rayleigh number (Ra).

It is noteworthy to mention that, when nanosized particles (single or more than two various materials) are suspended (at a lower concentration) along with the motile microorganisms in the host liquid, the thermal conducting property of the carrier fluid is improved, which leads to the enhanced microorganism transport and heat transport characteristics^25–29^. In fact, the transport process is further modulated in the presence of magnetizing fields (applied externally), which generates Lorentz force due to the interaction with the electrically conducting liquid. Such, an effect is very effective for controlling the transport process in a precious manner pertaining to nano-bio-technology. The above topic is very popular in drug delivery systems, tumor treatment, pharmacodynamics, hemodynamics, bacteria-powered micro-mixers, bio-energy systems, biological polymer blending, and others^4,30–36^. There are few pioneering works on the MHD mixed bioconvection involving oxytactic microorganisms. In this regard, Sheremet et al.^37^ studied MHD buoyant convective dynamics in an oblique enclosure in presence of nanofluid and gyrotactic microorganisms and found that an rise in the intensity of magnetic field causes the suppression of both heat and mass transfer. Later, MHD oxytactic bioconvection in a porous cavity with sidewall heated and cooled sliding top geometry has been scrutinized by Ahmed et al.^38^. They observed that applied magnetic field strength alters the bioconvective process. Mogharrebi et al.^39^ studied the mixed MHD oxytactic bioconvection of nanofluidic flow over a rotating cone and observed that oxytactic microorganisms concentration, as well as temperature pattern, is affected by the magnetic field intensity. In a recent study, Biswas et al.^40^ scrutinized the MHD bioconvective phenomena in a sidewall moving porous enclosure heated with a rounded bottom surface and packed with water based nanoparticles and oxytactic microorganisms. They found that the oxygen and microorganisms density is influenced by the bottom wall curvature, moving wall velocity, and magnetic field strength. The same group has also found that magnetic field intensity, speed, and direction of the moving walls alter the MHD thermo-bioconvection in a differentially heated porous enclosure with moving horizontal adiabatic walls and filled with oxytactic microorganisms^41^. Bég et al.^42^ analyzed the mixed bioconvective phenomena in the presence of oxytactic bacteria and nanofluidic flow over a porous layer over the vertical plate and found that the transport process is significantly controlled by the buoyancy ratio; increase in mixed convection parameters suppresses the motile microorganism density and nanoparticle concentration. Considering the oxytactic microorganisms, free-bioconvective phenomena in the presence of nanofluid filled porous structure have also been investigated by several researchers without^43–45^ and with^46^ magnetic fields. Furthermore, some of the researchers have also studied the nanofluidic mixed bioconvection in the presence of stimulant like gravity without porous media over circular cylinder^47^, with porous media over solid sphere^48^. The mixed bioconvection has also been conducted under the influence of a magnetic field and nanofluid flow over a rotating sphere^49^, lid-driven porous cavity^50^, etc. The mixed bioconvective process has been investigated in presence of magnetic field^51^, in absence of magnetic field^52,53^.

Apart from the mixed bioconvective phenomena, numerous researchers have inspected the nanofluidic mixed convective phenomena in various geometries. In this context, Abu-Nada and Chamkha^54^ analysed nanofluidic mixed thermal convection in a bottom cold wavy walled cavity with a moving hot wall at the top. Considering nanofluid flow in a wavy heated bottom and side cold with moving top has been scrutinized by Azizul et al.^55^ and Pal et al.^56^. Alsabery et al.^57^ have done the study of the mixed nanofluidic convection in a differentially heated cavity with moving sidewalls with the top wavy wall. All these studies have demonstrated the effect of undulation and speed of the moving wall on thermal convection. The study has also been extended further considering magnetic field^58,59^, porous substance^60^, and others^61–68^.

Searching the vast pool of existing literature, it shows that the bioconvective heat and mass transfer phenomena in a complex thermo-fluid flow system are a promising field in modern medical science and nano-biotechnology processes. In fact, suspension of live species (like microorganisms), as well as solid nano-size particles, facilitates for design several of nanoscale devices, which have practical applications as well. Although several researchers have conducted bioconvection phenomena in various geometry involving MHD, nanoparticles, and different stimuli (like light, gravity, oxygen, or chemical attraction, etc.); the transport process in a system involving triple convective phenomena, irregular geometry, and boundary conditions are abundant. To the best of the authors’ knowledge, mixed thermo-bioconvection of magnetically susceptible fluid containing copper nanoparticles and oxytactic bacteria in a W-shaped porous cavity is not comprehensive enough. In fact, the analysis of such convective dynamics is a rather complex phenomenon.

Therefore, the novelty of the present investigation is to combine the effect of the shear force (induced by the top sliding wall), buoyant force (induced by the bottom heat source), bioconvective flow (due to suspension of oxytactic bacteria or organisms) and Lorentz force (due to the imposed magnetic field) in the presence of Cu-water nanofluid-saturated porous substance. Therefore, this study aims to explore the mixed thermo-bioconvection of magnetically susceptible fluid containing copper nanoparticles and oxytactic bacteria in a novel W-shaped porous cavity. The buoyant convention is generated due to the isothermal heating at the wavy bottom wall, whereas the mixed convection is induced due to the shearing motion of the top-cooled sliding wall. The inclined sidewalls are insulated. The geometry is packed with Cu-water nanofluid-saturated porous structure, which is subjected to a magnetizing field acted horizontally. The study is carried out extensively by combining the influence of geometric parameters such as number (*m*) and amplitude (*δ*) of the bottom waviness, sidewall inclination (*γ*), and flow control parameters such as bioconvection Rayleigh number (R_b_), Reynolds number (Re), Grashof number (Gr), Peclet number (Pe), Lewis number (Le), oxygen diffusion ratio ($\chi$), Darcy number (Da), and Hartmann number (Ha). Finally, the various effects on the thermo-fluid flow, heat, and mass transfer characteristics are analyzed and elaborated with a physical explanation. The outcome of this study has intense potential applications in micro-mixers driven by bacteria, biological polymer production, drug delivery progressions, tumor treatment, hemodynamics, pharmacodynamics, bio-energy systems, bio-microsystems, microbial enhanced oil recovery, pollutant dispersion in aquifers, etc.^1–4^. All the figures/plots are generated in the MATLAB version 2019a software platform^69^.

## Flow model descriptions

**Figure 1:**
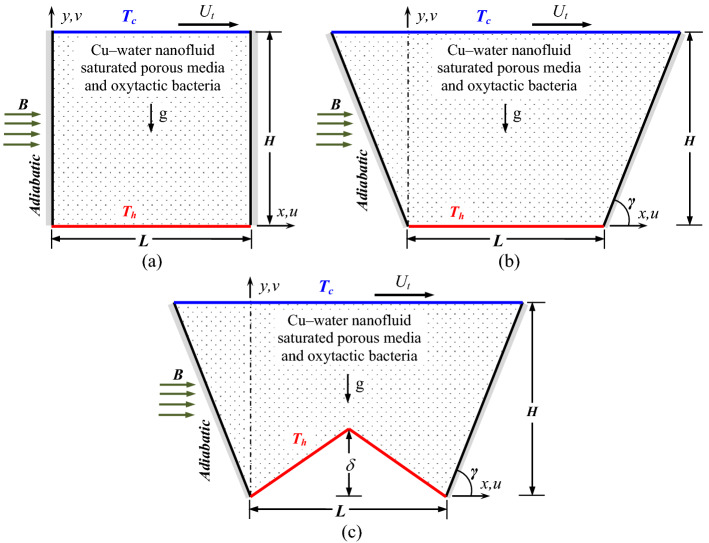
Schematics of the problem geometry including boundary conditions with (**a**) square cavity, (**b**) trapezoidal cavity, and (**c**) W-shaped cavity.

The chosen problem involves triple convective phenomena, irregular geometry, and boundary conditions. As a first step in the problem modeling, a two-dimensional classical square enclosing container with dimensions of *H* (height) and *L* (length) is taken, which is elucidated in Fig. 1a. The bottom horizontal wall is subjected to a constant heat source (at a temperature *T*_*h*_); while the top horizontal wall is cooled (*T*_*c*_ < *T*_*h*_) translating at a constant speed (*U*_*t*_) along the right side. The inclined sidewalls are thermally insulated. The geometry is filled with porous structure and water based Cu nanoparicles, which is subjected to a magnetizing field acted horizontally. The enclosure is filled with Cu–H_2_O nanofluid (of volumetric concentration, *χ*), porous structure (with a uniform porosity, $\varepsilon$), and oxytactic bacteria, acted horizontally by a uniform magnetic field having intensity *B*. Keeping the same bottom heating length (*L* = *H*), the square cavity is reformed into a trapezoidal cavity (by increasing the top moving cold wall length), as elucidated in Fig. 1b. Here, the sidewalls are orientated at an angle *γ* with the base. Furthermore, the bottom heated wall is modified into a triangular shape with a number (*m*) and amplitude (*δ*) of the undulations, which is depicted in Fig. 1c. The existence of a horizontal uniform magnetic field (with intensity *B*) in the enclosure generates Lorentz force due to the mutual interaction of the imposed magnetic field and nanofluid (through its electrical conducting characteristics). The Lorentz force counteracts the buoyant force. The magnetic field intensity is controlled through the Hartmann number (Ha). Furthermore, the influence of sliding wall motion is controlled by the Reynolds number (Re); whereas convective strength is assessed through the Grashof number (Gr).

Here, it is supposed that porous material is non-deformable and homogenous in nature and is saturated with water-based Cu nanoparticles and oxytactic microorganisms^22^. The Forchheimer–Brinkman-extended Darcy model is followed in modeling the porous layers adopting the local thermodynamic equilibrium approach^17,70,71^. It is anticipated that size of the oxytactic bacteria is much smaller than the pores of the porous structure and therefore oxytactic bacteria are not absorbed by the porous layers. Furthermore, it is assumed that the presence of the porous structure and inclusion of the nanopowders has no impact on the swim velocity of the blended microorganisms^4,38,39^. The modeling of the bioconvective transport phenomena involving oxytactic microorganisms is adopted following the continuum model of Hillesdon and Pedley^5^. The change in fluid density due to the alternation of fluid temperature is handled by the Boussinesq approximation. For the analysis of the governing flow models, the nanofluidic flow along with the suspended microorganisms through the porous layers is supposed to be laminar, steady, incompressible, and Newtonian. In fact, the radiation effects and viscous dissipation has negligible contribution compared to the conduction and convection^17^. The influence of Joule heating, Hall effect and magnetic field induction are also insignificant^4,72^. The no-slip condition and impermeable state of the solid walls are considered^73^. Based on these considerations, the evolved continuity, momentum, energy, oxygen, and cell conservation equations are expressed as^4,22,37^

1 $$\frac{\partial u}{{\partial x}} + \frac{\partial v}{{\partial y}} = 0$$

2 $$\frac{1}{{\varepsilon^{2} }}\left( {u\frac{\partial u}{{\partial x}} + v\frac{\partial u}{{\partial y}}} \right) = - \frac{1}{\rho }\frac{\partial p}{{\partial x}} + \frac{\nu }{\varepsilon }\left( {\frac{{\partial^{2} u}}{{\partial x^{2} }} + \frac{{\partial^{2} u}}{{\partial y^{2} }}} \right) - \frac{\nu }{K}u - \frac{{F_{c} }}{\sqrt K }u\sqrt {u^{2} + v^{2} }$$

3 $$\begin{aligned} \frac{1}{{\varepsilon^{2} }}\left( {u\frac{\partial v}{{\partial x}} + v\frac{\partial v}{{\partial y}}} \right) &= - \frac{1}{\rho }\frac{\partial p}{{\partial y}} + \frac{\nu }{\varepsilon }\left( {\frac{{\partial^{2} v}}{{\partial x^{2} }} + \frac{{\partial^{2} v}}{{\partial y^{2} }}} \right) - \frac{\nu }{K}v - \frac{{F_{c} }}{\sqrt K }v\sqrt {u^{2} + v^{2} } - \frac{1}{\rho }\sigma B^{2} v \hfill \\ \, & \quad + \frac{1}{\rho }\left[ {\gamma \Delta \rho \cdot n - \rho \beta \left( {T - T_{c} } \right)} \right]g \hfill \\ \end{aligned}$$

4 $$u\frac{\partial T}{{\partial x}} + v\frac{\partial T}{{\partial y}} = \frac{{\varepsilon k + (1 - \varepsilon )k_{ps} }}{{(\rho C_{P} )}}\left( {\frac{{\partial^{2} T}}{{\partial x^{2} }} + \frac{{\partial^{2} T}}{{\partial y^{2} }}} \right)$$

5 $$u\frac{\partial C}{{\partial x}} + v\frac{\partial C}{{\partial y}} = D_{c} \left( {\frac{{\partial^{2} C}}{{\partial x^{2} }} + \frac{{\partial^{2} C}}{{\partial y^{2} }}} \right) - \lambda n$$

6 $$\frac{\partial }{\partial x}\left[ {un + \tilde{u}n - D_{n} \frac{\partial n}{{\partial x}}} \right] + \frac{\partial }{\partial y}\left[ {vn + \tilde{v}n - D_{n} \frac{\partial n}{{\partial y}}} \right] = 0$$

The modeling of the transport equation Eqs. (2) and (3) are carried out following the Forchheimer–Brinkman-extended Darcy model, where the term *F*_c_ ${(} = {1}{\text{.75/}}\sqrt {{150}\varepsilon^{{3}} } {)}$ is known as the Forchheimer coefficient (which is the inertial friction), and *K* $( = \varepsilon^{3} d_{ps}^{2} /150(1 - \varepsilon )^{2} )$ is the porous substance permeability, *ε* signifies porosity*,* and *d*_ps_ is porous structure particles size^17^. In this study, both the flow medium $(k)$ and solid porous phase $(k_{ps} )$ thermal conductivities are also taken into consideration in Eq. (4). Moreover, the thermal conductivity (effective) of the nanofluid-saturated porous substance is taken into consideration using the term $(\varepsilon k + (1 - \varepsilon )k_{ps} )$ following the local thermal equilibrium approach^17^. The microorganisms average swimming speed ($\tilde{u}$ and $\tilde{v}$) are calculated as^5^

7 $$\tilde{u} = \left( {\frac{{bW_{c} }}{\Delta C}} \right)\frac{\partial C}{{\partial x}}{\text{ and }}\tilde{v} = \left( {\frac{{bW_{c} }}{\Delta C}} \right)\frac{\partial C}{{\partial y}}$$

where $W_{c}$ is the cell swimming velocity (maximum), *b* is the fixed parameter, $C_{0}$ is the wall oxygen concentration (and thus $\Delta C = C_{0} - C_{\min }$). In fact, for the microorganisms to be alive, there is a requirement of minimum oxygen concentration (expressed by the term $C_{\min }$), $- \lambda n$ which indicates the utilization of oxygen by the microorganism.

Now, the dimensional transport equations are transformed into nondimensional form (in Cartesian coordinates) as^4,22,23,44,74^

8 $$\frac{\partial U}{{\partial X}} + \frac{\partial V}{{\partial Y}} = 0$$

9 $$\frac{1}{{\varepsilon^{2} }}\left( {U\frac{\partial U}{{\partial X}} + V\frac{\partial U}{{\partial Y}}} \right) \, = \, - \frac{\partial P}{{\partial X}} + \, \frac{1}{{\varepsilon {\text{Re}}}}\frac{\nu }{{\nu_{f} }}\left( {\frac{{\partial^{2} U}}{{\partial X^{2} }} + \frac{{\partial^{2} U}}{{\partial Y^{2} }}} \right) \, - \left( {\frac{\nu }{{\nu_{f} }}\frac{{1}}{{{\text{DaRe}}}} + \frac{{F_{c} \sqrt {U^{2} + V^{2} } }}{{\sqrt {{\text{Da}}} \, }}} \right){\kern 1pt} U$$

10 $$\begin{gathered} \frac{1}{{\varepsilon^{2} }}\left( {U\frac{\partial V}{{\partial X}} + V\frac{\partial V}{{\partial Y}}} \right) \, = \, - \frac{\partial P}{{\partial Y}} + \, \frac{1}{{\varepsilon {\text{Re}}}}\frac{\nu }{{\nu_{f} }}\left( {\frac{{\partial^{2} V}}{{\partial X^{2} }} + \frac{{\partial^{2} V}}{{\partial Y^{2} }}} \right) - \left( {\frac{\nu }{{\nu_{f} }}\frac{{1}}{{{\text{DaRe}}}} + \frac{{F_{c} \sqrt {U^{2} + V^{2} } }}{{\sqrt {{\text{Da}}} \, }}} \right){\kern 1pt} V \hfill \\ \, - \frac{{\rho_{f} }}{\rho }\frac{\sigma }{{\sigma_{f} }}\frac{{{\text{Ha}}^{2} }}{{{\text{Re}}}}V + \frac{(\rho \beta )}{{\rho \beta_{f} }}\frac{{{\text{Gr}}}}{{{\text{Re}}^{{2}} }}(\theta - {\text{R}}_{{\text{b}}} N) \hfill \\ \end{gathered}$$

11 $$U\frac{\partial \theta }{{\partial X}} + V\frac{\partial \theta }{{\partial Y}} = \frac{1}{{{\text{RePr}}}}\frac{\alpha }{{\alpha_{f} }}\left( {\frac{{\partial^{2} \theta }}{{\partial X^{2} }} + \frac{{\partial^{2} \theta }}{{\partial Y^{2} }}} \right)$$

12 $$U\frac{\partial \zeta }{{\partial X}} + V\frac{\partial \zeta }{{\partial Y}} = \frac{1}{{{\text{ReLePr}}}}\left( {\frac{{\partial^{2} \zeta }}{{\partial X^{2} }} + \frac{{\partial^{2} \zeta }}{{\partial Y^{2} }}} \right) - \frac{{\sigma_{1} }}{{{\text{ReLePr}}}}N$$

13 $$\begin{gathered} \chi \left( {U\frac{\partial N}{{\partial X}} + V\frac{\partial N}{{\partial Y}}} \right) = \frac{1}{{{\text{ReLePr}}}}\left( {\frac{{\partial^{2} N}}{{\partial X^{2} }} + \frac{{\partial^{2} N}}{{\partial Y^{2} }}} \right) \hfill \\ \, - \frac{{{\text{Pe}}}}{{{\text{ReLePr}}}}\left( {N\frac{{\partial^{2} \zeta }}{{\partial X^{2} }} + N\frac{{\partial^{2} \zeta }}{{\partial Y^{2} }} + \frac{\partial N}{{\partial X}}\frac{\partial \zeta }{{\partial X}} + \frac{\partial N}{{\partial Y}}\frac{\partial \zeta }{{\partial Y}}} \right) \hfill \\ \end{gathered}$$

During the transformation of the dimensional equations into nondimensional, the following scale factors are introduced

14 $$\begin{gathered} (X,Y) = (x,y)/H \,\,\,\,\, (U,V) = (u,v)/U_{t} \,\,\,\,\,\, P = (p - p_{a} )/\rho U_{t}^{2} \, \hfill \\ \theta = (T - T_{c} )/(T_{h} - T_{c} ) \,\,\,\,\, \zeta = (C - C_{\min } )/\Delta C \,\,\,\,\,\, N = n/n_{o} \hfill \\ \end{gathered}$$

where *U* and *V* are the components of velocity (dimensionless) along with the *X* and *Y* directions, *P* is the pressure, *θ* is the temperature, $\zeta$ is the oxygen concentration, and *N* is the microorganisms number density. Furthermore, following dimensionless parameter are also yields during the transformation of the dimensional governing equations

15 $$\begin{gathered} {\text{R}}_{{\text{b}}} = \frac{{n_{o} (\rho_{cell} - \rho_{f} )\omega }}{{\rho_{f} \beta_{f} (T_{h} - T_{c} )}},\;{\text{ Pe}} = \frac{{bW_{c} }}{{D_{n} }}, \, \;{\text{Le}} = \frac{{\alpha_{f} }}{{D_{{\text{c}}} }}, \, \quad {\text{Pr}} = \frac{{\nu_{f} }}{{\alpha_{f} }}\;\; \hfill \\ {\text{Gr}} = \frac{{g\beta_{f} (T_{h} - T_{c} )H^{3} }}{{\nu_{f}^{2} }},\;\;{\text{Re}} = \frac{{U_{t} H}}{{\nu_{f} }},\quad {\text{Ri}} = \frac{{{\text{Gr}}}}{{{\text{Re}}^{{2}} }},\quad {\text{Da}} = \frac{K}{{H^{{2}} }}, \hfill \\ {\text{Ha}} = BH\sqrt {\frac{{\sigma_{f} }}{{\mu_{f} }}} , \, \sigma_{1} = \frac{{n_{o} \lambda H^{2} }}{{D_{c} \Delta C}},\; \, \chi = \frac{{D_{c} }}{{D_{n} }} \hfill \\ \end{gathered}$$

where, the dimensionless variables Pr, Re, Da, Ha, Gr, Ri, R_b_, Le, Pe are the Prandtl, Reynolds, Darcy, Hartmann, Grashof, Richardson, bioconvective Rayleigh, Lewis, and Peclet numbers, respectively. *χ* is the diffusion ratio.

Therefore, the dimensionless transport Eqs. (8)–(13) are solved numerically by employing the correct boundary conditions, as summarized in Table 1.

**Table 1:** Conditions of the boundaries for the flow model^22,38^, where ‘*a*’ indicates normal direction to the surface.

Boundary	Velocity conditions	Temperature and concentration conditions
Bottommost heated wall	$U = V = 0$	$\theta = {1,} \; \zeta = {1},{\text{ Pe}} \, N{\kern 1pt} \, \partial \zeta /\partial a = \partial N/\partial a$
Side walls	$U = 0, \, V = 0$	$\partial \theta /\partial a = 0, \, \zeta = N = {1}$
Top cold moving wall	$U = 1, \, V = 0$	$\theta = {0,} \; \partial \zeta /\partial a = \, \partial N/\partial a = {0}$

As the base liquid is a mixture of water (with fixed Pr = 5.83), and suspension of Cu nanoparticles, the effective properties^75^ of nanofluid (like density, thermal expansion coefficient, and specific heat) are computed using the appropriate correlations^4^ as listed in Tables 2 and 3. The symbols *φ* and *f* designate nanoparticle concentration (volumetric) and base fluid (water).

**Table 2:** Effective properties of nanoparticles and base fluid^75^.

Properties	Cu	Water
$\mu$ (kg m^−1^ s^−1^)	–	9.09 × 10^–4^
$\rho$ (kg m^−3^)	8933	997.1
$\beta$ (K^−1^)	1.67 × 10^–5^	21 × 10^–5^
$C_{p}$ (Jkg^−1^ K^−1^)	385	4179
$K$ (Wm^−1^ K^−1^)	401	0.613

**Table 3:** Correlations for the properties (effective) of Cu-water nanofluid^4^.

Effective parameters	Relations
Density,$\rho$	$\rho = \, (1 - \varphi )\rho_{f} \, + \, \varphi \, \rho_{{\text{s}}}$
Thermal conductivity,$k$	$k \, = \, k_{f} \left[ {\frac{{(k_{{\text{s}}} \, + \, 2k_{{\text{f}}} ) - 2\varphi (k_{{\text{f}}} - k_{{\text{s}}} )}}{{(k_{{\text{s}}} \, + \, 2k_{{\text{f}}} ) + \varphi (k_{{\text{f}}} - k_{{\text{s}}} )}}} \right]$
Specific heat capacity,$(\rho c_{p} )$	$(\rho c_{p} ) = (1 - \varphi )(\rho c_{p} )_{f} + \, \varphi {(}\rho c_{p} {)}_{{\text{s}}}$
Thermal expansion coefficient,$(\rho \beta )$	$(\rho \beta ) = (1 - \varphi )(\rho \beta )_{f} + \, \varphi { (}\rho \beta {)}_{{\text{s}}}$
Electrical conductivity,$\sigma$	$\sigma \, = \, \sigma_{f} \left[ {1 + \frac{{3(\sigma_{s} {/}\sigma_{f} - 1)\varphi }}{{(\sigma_{s} {/}\sigma_{f} + 2) - (\sigma_{s} {/}\sigma_{f} - 1)\varphi }}} \right]$
Thermal diffusivity,$\alpha$	$\alpha = \frac{k}{{(\rho c_{p} )}}$
Viscosity,$\mu$	$\mu \, = \, \frac{{\mu_{f} }}{{(1 - \varphi )^{2.5} }}$

After conducting the computations, the stored data are post-processed for evaluating the local Nusselt (Nu_loc_) and Sherwood numbers (Sh_loc_) over the heated walls following expression as

16a $${\text{Nu}}_{{{\text{loc}}}} = \frac{k}{{k_{f} }}\left( { - \left. {\frac{\partial \theta }{{\partial M}}} \right|_{{\text{curved wall}}} } \right)\;\;{\text{and}}\;\;{\text{Sh}}_{{{\text{loc}}}} = \left( { - \left. {\frac{\partial \zeta }{{\partial M}}} \right|_{{\text{curved wall}}} } \right)$$

Further integrating the above Eq. (16a), the average Nusselt (Nu) and Sherwood numbers (Sh) are also evaluated

16b $${\text{Nu}} = \frac{k}{{k_{f} }}\frac{1}{S}\int\limits_{0}^{S} {\left( { { - {\frac{\partial \theta }{{\partial M}}} {\Big |_{{\text{curved wall}}}} } } \right)} \, dS\;\;{\text{and}}\;\;{\text{Sh}} = \frac{1}{S}\int\limits_{0}^{1} {\left( { { - {\frac{\partial \zeta }{{\partial M}}} {\Big |_{{\text{curved wall}}}} } } \right)dS}$$

where the symbols ‘*S*’ and ‘*M*’ correspond to the definite distance over the curvature and the perpendicular direction of the curvature (as depicted in Fig. 1). Finally, the local distribution of velocity, static temperature, concentrations of oxygen as well as microorganisms are elucidated through streamlines ($\psi$), isotherms ($\theta$), isoconcentrations of oxygen ($\zeta$), and microorganisms (*N*). It is pertinent to mention that, the streamlines are generated by solving the function ($\psi$)

17 $$- \frac{\partial \psi }{{\partial X}} = V\;\;{\text{and}}\;\;\frac{\partial \psi }{{\partial Y}} = U$$

## Solutions methodology

The finite volume method-based FORTRAN code is developed to solve the nondimensional partial differential Eqs. (8)–(13) applying the proper conditions of the boundaries. The discretization of the computational domain is carried out through the appropriate distribution of nonuniform staggered grids (allotting finer mesh over the boundary). Thereafter the discretization helps to convert the equations into a nonlinear algebraic form, which are solved iteratively following the SIMPLE algorithm^71,76^, ADI sweep, and TDMA. For obtaining the converged results after the correct computations, the residuals and mass-defect criteria are limited to < 10^–8^ and 10^–10^ respectively.

The developed code has been validated extensively with the in-house experimental results^77^ as well as published results considering complex flow situations (like nanofluid, porous substance, magnetic field, microorganism)^4,78–81^. Further to the above, a validation study with thermo-bioconvection dynamics with the suspended oxytactic bacteria is also conducted by simulating the problem of published work^22,74^. The problem is a differentially heated cavity consisting of a porous structure and oxytactic microorganisms. From the computed results, the average Nusselt (Nu) and Sherwood number (Sh) at the active wall is computed, which are depicted in Table 4, which shows excellent matching with both the results. It confirms the accuracy of the present solver.

**Table 4:** Comparative assessment of the thermo-bioconvection in-between the present result with published results^22,74^ using average values of Nu and Sh when Ra = 10, Pr = 1, Le = 1.

Ra	R_b_	Le	Pe	Nusselt number (average)			Sherwood number (average)		
				Present results	Sheremet and Pop^22^	Hussain et al.^74^	Present results	Sheremet and Pop^22^	Hussain et al.^74^
10	10	1	0.1	1.0817	1.0775	1.0774	0.3369	0.3368	0.3368
	100	1	0.1	1.0761	1.0717	1.0716	0.3444	0.3447	0.3446
100	10	1	0.1	3.0967	3.0910	3.0915	0.2522	0.2506	0.2504

For capturing the correct results involving complex geometry under the multiphysical scenario, the selection of the appropriate grids is very important. For this purpose, a grid independency test is carried out for the various grid sizes of 320 × 60, 360 × 100, 400 × 140, and 440 × 180. The effects of the mesh size are compared by the average Nu on the heated bottom wall for the varying R_b_ = 0 to 100 keeping Le = 1, Pe = 1, $\chi$ = 1, Re = 100, Gr = 10^4^, Da = 10^–3^, $\varepsilon$ = 0.8, $\varphi$ = 2%, Ha = 30, *m* = 1, *δ* = 0.3 and *γ* = 60°, fixed. In fact, finer grids are distributed closer to the curved walls for capturing correct hydrodynamics and change in the concentration gradients under the various controlling parameters. The evaluated average Nu along with the error estimation (in %) are shown in Table 5, which shows < 1% errors with 400 × 140 grid size beyond which the changes in the average Nu are minor. Therefore, the grids with 400 × 140 mesh are selected for the extensive computations.

**Table 5:** Grid sensitiveness with R_b_ = 0 to 100, Le = 1, Pe = 1, $\chi$ = 1, Re = 100, Gr = 10^4^, Da = 10^–3^, $\varepsilon$ = 0.8, $\varphi$ = 2%, Ha = 30, *m* = 1, *δ* = 0.3 and *γ* = 60°.

R_b_	Average Nusselt number (consecutive error in %)			
	320 × 60	360 × 100	400 × 140	440 × 180
1	4.683	4.613 (1.52%)	4.593 (0.44%)	4.591 (0.04%)
10	4.775	4.651 (2.67%)	4.620 (0.67%)	4.617 (0.06%)
50	4.829	4.712 (2.47%)	4.678 (0.74%)	4.672 (0.13%)
100	4.604	4.475 (2.87%)	4.434 (0.94%)	4.427 (0.16%)

## Results and discussion

In this study, a hydro-magnetic mixed thermo bio-convection in a novel W-shaped porous enclosure, filled with Cu-water nanofluid and oxytactic microorganisms is examined numerically. This porous enclosure is bottom heated, has adiabatic sidewalls, and cold top wall having right directional sliding velocity. The bio-convection process of Cu-water nanofluid with oxytactic microorganisms is adjusted by the external magnetic field, acts horizontally. This W-shaped geometry is assumed to be a modified form of a square geometry. The top wall surface of square geometry is raised for becoming a trapezoidal enclosure keeping the same bottom wall length. Next, the bottom surface is modified adopting a triangular undulated surface that leads to form our considered W-shaped cavity. Therefore, this shaped enclosure raises the cooling and heating area, the corresponding change in the working flow volume accordingly. It results in the impact on flow, heat transfer, and mass transfer in the enclosure. The impact of cooling length is addressed by altering side angles, the heating length is varied by amplitudes (*δ* = 0–0.4) and number of undulation (*m* = 0–4). Apart from the particular effect of the shape of the enclosure, the other controlling parameters like bioconvective Rayleigh number (R_b_ = 0–100), Peclet number (Pe = 0.1–5.0), Lewis number (Le = 0.1–5.0), porous substance permeability (Da = 10^–4^–10^–1^), magnetizing field strength (Ha = 0–70), and Grashof number (Gr = 10–10^4^) are also varied. The outcome is presented by flow field ($\psi$), isotherms ($\theta$), oxygen ($\zeta$) and microorganisms (*N)* isoconcentrations in this study. The systematic study of bio-convection is presented in the subsequent sections through the effect of a) different shapes (square, trapezoidal, W-shaped cavity) with R_b_, b) amplitude (*δ*), c) number of undulations (*m*), d) sidewall inclination angle (*γ*), e) varying Da and Ha, f) varying Le, and Pe, and g) varying Gr keeping the fixed values of Re = 100, *ε* = 0.8, $\chi$ = 1, and $\varphi$ = 2%.

### Influence of different shapes and bioconvection Rayleigh number (R_b_)

**Figure 2:**
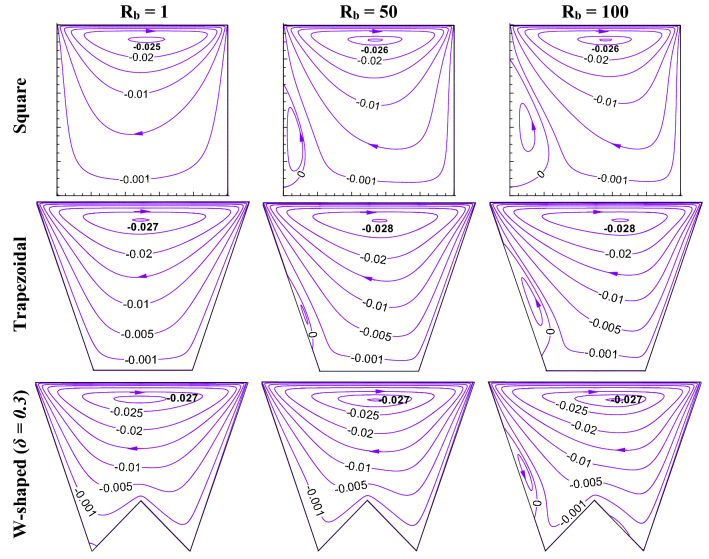
Streamline contours ($\psi$) for different geometric cavity at Le = Pe = 1, Gr = 10^4^, Da = 10^–3^, Ha = 30 for square, trapezoidal and W-shaped cavities.

**Figure 3:**
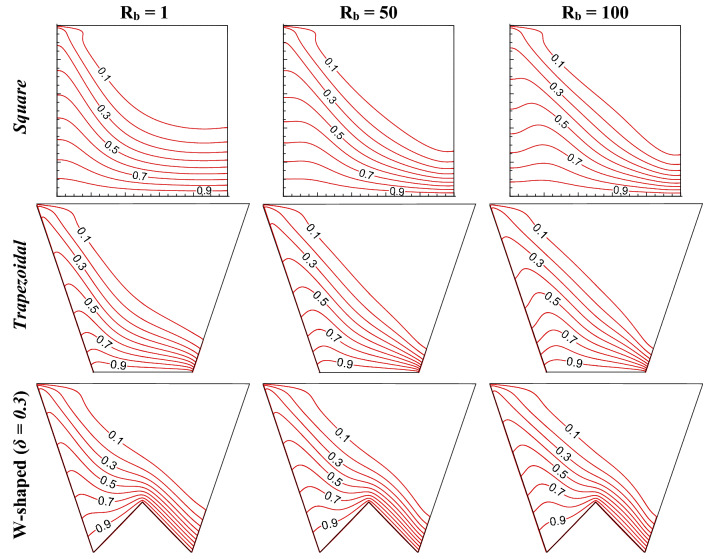
Isotherms contours ($\theta$) for different geometric cavity at Le = Pe = 1, Gr = 10^4^, Da = 10^–3^, Ha = 30 for square, trapezoidal and W-shaped cavities.

**Figure 4:**
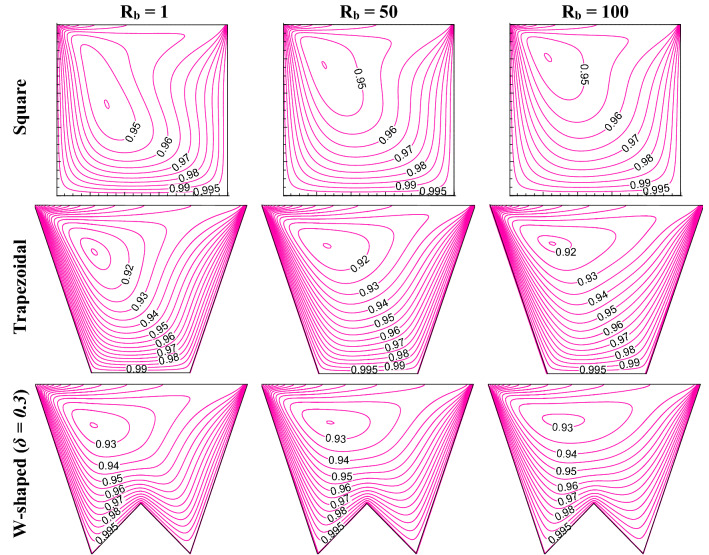
Oxygen isoconcentration ($\zeta$) for different geometric cavity at Le = Pe = 1, Gr = 10^4^, Da = 10^–3^, Ha = 30 for square, trapezoidal and W-shaped cavities.

**Figure 5:**
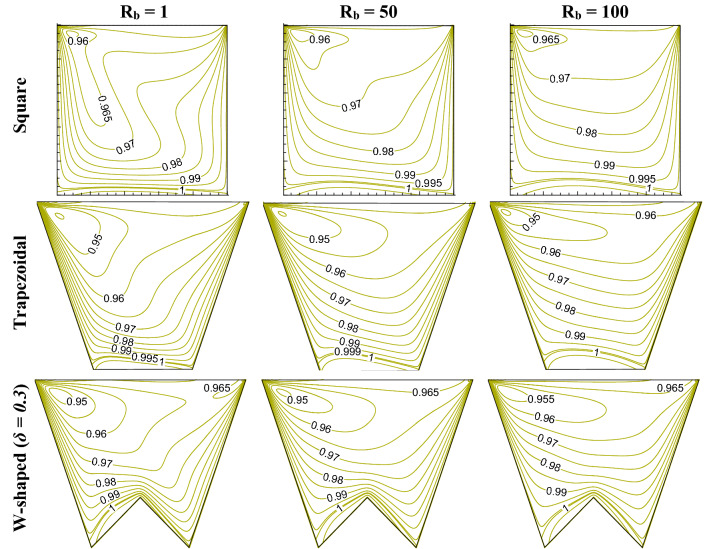
Microorganisms isoconcentration (*N*) for different geometric cavity at Le = Pe = 1, Gr = 10^4^, Da = 10^–3^, Ha = 30 for square, trapezoidal and W-shaped cavities.

This section presents the influence of different shapes (square, trapezoidal, W shaped cavity) and bioconvective Rayleigh number (R_b_) on bioconvection process by flow structure (in Fig. 2), static temperature (in Fig. 3), oxygen isoconcentrations (in Fig. 4) and microorganisms isoconcentrations (in Fig. 5). The reason for choosing these types of shapes could be understood easily from the effect geometric design analysis point of view. Of course, a classical square enclosure means equal heating (*L*_*h*_) and cooling (*L*_*c*_) surface, the cooling surface rises for trapezoidal geometry with a corresponding rise in working fluid volume (*V*_*f*_), and W-shaped geometry raises the heating surface by reducing the working flow volume. The present comparative study of their shapes is the stepwise evolution of the W-shape that may depict the influence of heating surface, cooling surface, and flow volume individually or combined. In fact, the values of R_b_ controls the buoyant force estimation owing to both thermal gradient and motility of microorganisms therefore, R_b_ can control the thermal energy transport with microorganisms’ population. To understand the effect, this results have been presented at Le = 1, Pe = 1, Da = 10^–3^, Gr = 10^4^, Ha = 30 for R_b_ of 1, 50 and 100 as in Figs. 2, 3, 4, 5).

Starting with a square cavity (first row), it is noted that the general buoyant force (due to the presence of temperature gradient) at the bottom of the cavity results in the fluid flow velocity, its direction is dictated by the sliding top cold wall. Thus, a single circulating cell clockwise occupies the whole space of the enclosure, as observed from Fig. 2 at R_b_ = 1.0. The center of the circulation lies at the top of the enclosure; streamlines are crowded adjacent to the top wall that leading to high flow strength due to the shear-driven velocity. Therefore, the depicted shear force owing to the moving wall has a great effect than that of buoyancy force. As R_b_ increases, the strength of the circulation rises though it is not significant; however, a secondary eddy (clockwise) is formed on the left adiabatic wall, and its size rises. The reason behind the formation of the secondary eddy is due to the impact of R_b_ which in turn produces counteracting force over the flow and helps the formation of the eddy. This finds a weak momentum location at the left wall. The reason for this zone is owing to the dominancy of shear-induced flow by the top wall that sucks/pulls the fluid from the lower wall to the upper left corner. Now, the streamlines for trapezoidal-shaped cavity (second row) show the impact of the rise in cooling surface (*L*_*c*_) as well as working fluid volume (*V*_*f*_) keeping the same heating surface (*L*_*h*_). The thermal energy transfer from the heated lower wall to the upper cold wall rises, rise in the shear-driven surface area develops more shear force. Formation of the secondary eddy is also noted at R_b_ = 50 and 100 like the square cavity, but its size is lesser relative to the square enclosure, this possibly owing to the lessening of a weak momentum zone near the left wall. From the streamlines of W-shaped geometry (third row), no significant improvement in streamlines’ strength is noted, rather small decrement is noted. A W-shaped cavity raises the heating surface (*L*_*h*_) that may produce more convection energy (for the same cooling surface as in a trapezoidal cavity); this effect is not reflected in the streamlines. The cause may be the reduction in the flowing fluid flow volume in the cavity. The chance of secondary eddy formation is also becoming less, as noted only at R_b_ = 100. Of course, due to the presence of lower undulation, streamlines are distorted in the lower portion of the W-shaped cavity.

The isotherms (first row) of the square enclosure (as in Fig. 3) illustrate that it cuts both adiabatic sidewalls, aligned diagonally from the right lower corner to the left wall. The pattern of isotherms over the left wall depicts the static temperature distribution, high to low (along the flow direction) over the whole wall, which in turn drops the temperature gradient from bottom to top, this causes the flow development over the left wall vertically. At the top wall, the fluid becomes cold due to the heat rejection and circulates (due to the shearing action of the top wall), which results in a cold zone at the right top corner. The right side bottom wall shows concentrated isotherms (thinner thermal boundary layers) that indicate a high heat transfer zone, the heat is carried away along the left direction because of the nature of the flow situation for the considered problem. The isotherms at the right bottom wall become clustered more as R_b_ rises and the pattern of isotherms at the left wall become wavy in nature. This wavy pattern indicates that the temperature of the fluid closure the adiabatic walls decreases and the nearby fluid temperature rises. This is due to the formation of a flow reversal zone due to an increase in R_b_ as noted from the streamlines. For the trapezoidal enclosure, the similar pattern of isotherms like square cavity and the trend with R_b_ is also similar. A significant change is noticed in the isotherms gradient. For a W-shaped cavity, the isotherms pattern changes its shape because of a triangular bottom wall. The isotherms gradient at the right side of the triangular base is more and highest at the apex of the triangle.

From the oxygen isoconcentrations (as in Fig. 4), it is observed that the Oxygen isoconcentrations form a balloon-shaped circulation targeting the top left corner of the square-shaped cavity. The oxygen isoconcentrations tend to move left top corner due to the combined effect of shear-induced flow and thermal convection. This circulation reduces its shape as R_b_ rises, which indicates more propagation of oxygen towards upward. A similar trend has been noted for trapezoidal-shaped and W-shaped cavities, while the circular shape becomes horizontal at the higher R_b_. Due to the high cooling effect, the oxygen isoconcentrations zone at the top wall is more for trapezoidal and W-shaped cavities. W-shaped cavity bottom heating shows a high gradient of oxygen isoconcentrations at the apex of the bottom triangle.

Microorganisms isoconcentrations swim toward the presence of oxygen population as noted in Fig. 5. Thus, the isoconcentrations move upward and concentrate on the left top corner, which is due to the shearing motion of the sliding wall. This uplift of microorganisms’ isoconcentrations is pronounced as R_b_ increases for all the cases of the square, trapezoidal and W-shaped cavities. Formation of the vortex of microorganisms isoconcentrations is also noted, its shape lessens with the rise in R_b_ value for all shapes considered. In fact, the microorganisms isoconcentrations stretched horizontally with the increasing R_b_.

**Figure 6:**
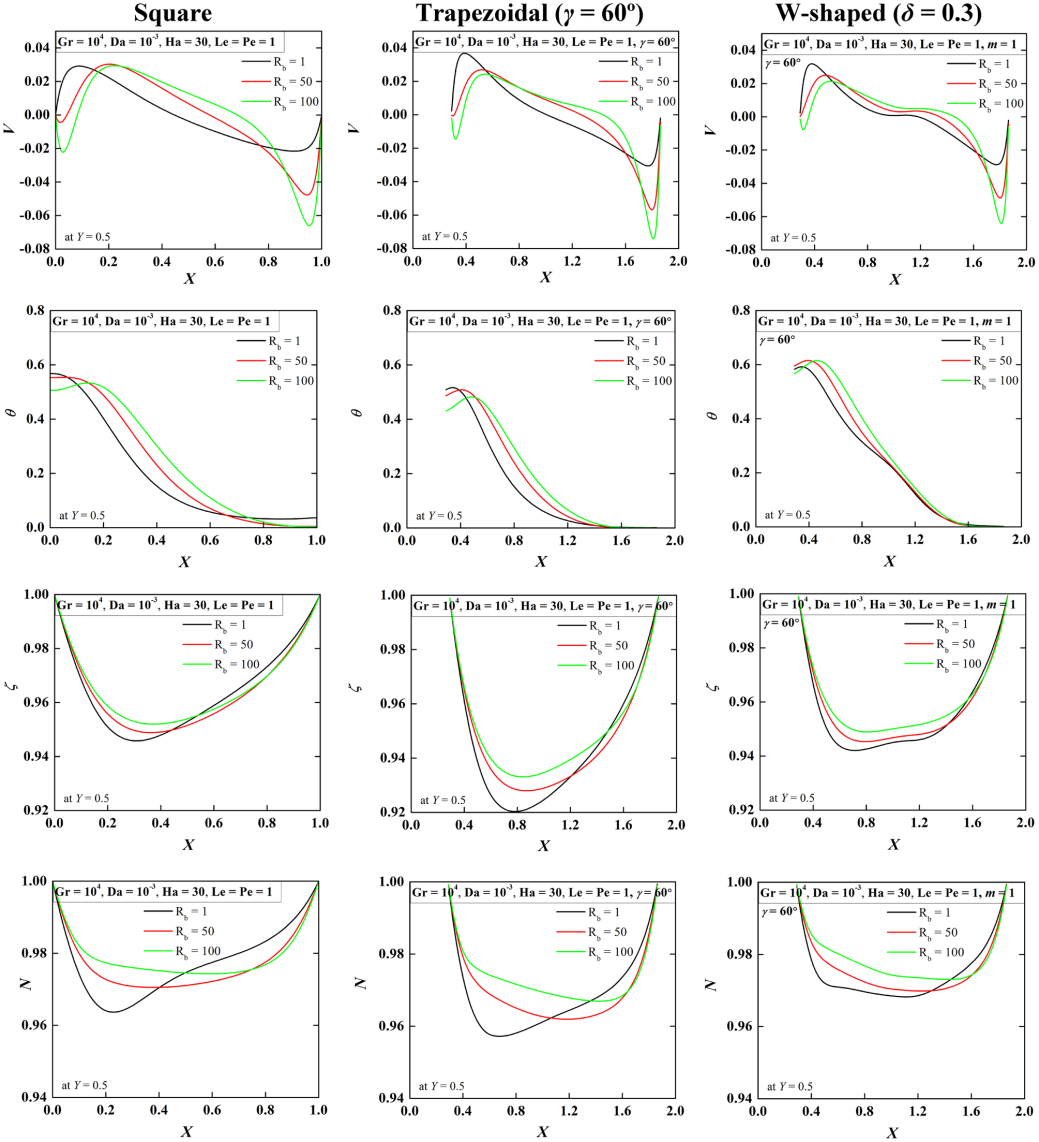
Distribution of local velocity (first-row), temperature (second-row), oxygen (third-row), and microorganisms (forth-row) for varying R_b_ = 1, 50, 100 at Le = Pe = 1, Gr = 10^4^, Da = 10^–3^, Ha = 30 for square (first column), trapezoidal (second column) and W-shaped (third column) cavities.

To understand insight of the nanofluidic flow physics in the enclosures, the local velocity, temperature, oxygen, microorganism isoconcentrations when R_b_ = 1, 50, 100 at Le = Pe = 1, Gr = 10^4^, Da = 10^–3^, Ha = 30 are illustrated in Fig. 6. The variables are chosen at the horizontal mid-plane (= 0.5*H*) of each cavity. The vertical velocity (first row) for the square cavity depicts the left side positive peak and right side negative peak velocity that indicates the clockwise flow circulation in the flow field. This has been noted in every case of streamlining presented. For the case of high R_b_, the left side small negative peak velocity is developed for the secondary vortex. This phenomenon is also observed in Fig. 2. Trapezoidal and W-shaped cavities show similar velocity patterns except for the magnitude. The temperature profiles (second row) for all the shapes show the high temperature at the left wall and the low temperature at the right wall. The developed thermal gradient leads to clockwise movement of flow. A wavy nature of the temperature profile is observed near the left wall at higher R_b_ due to the flow reversal phenomenon. Oxygen and microorganisms isoconcentrations show less value at the mid-region, this value rises with R_b_ for all considered cases of cavities.

**Figure 7:**
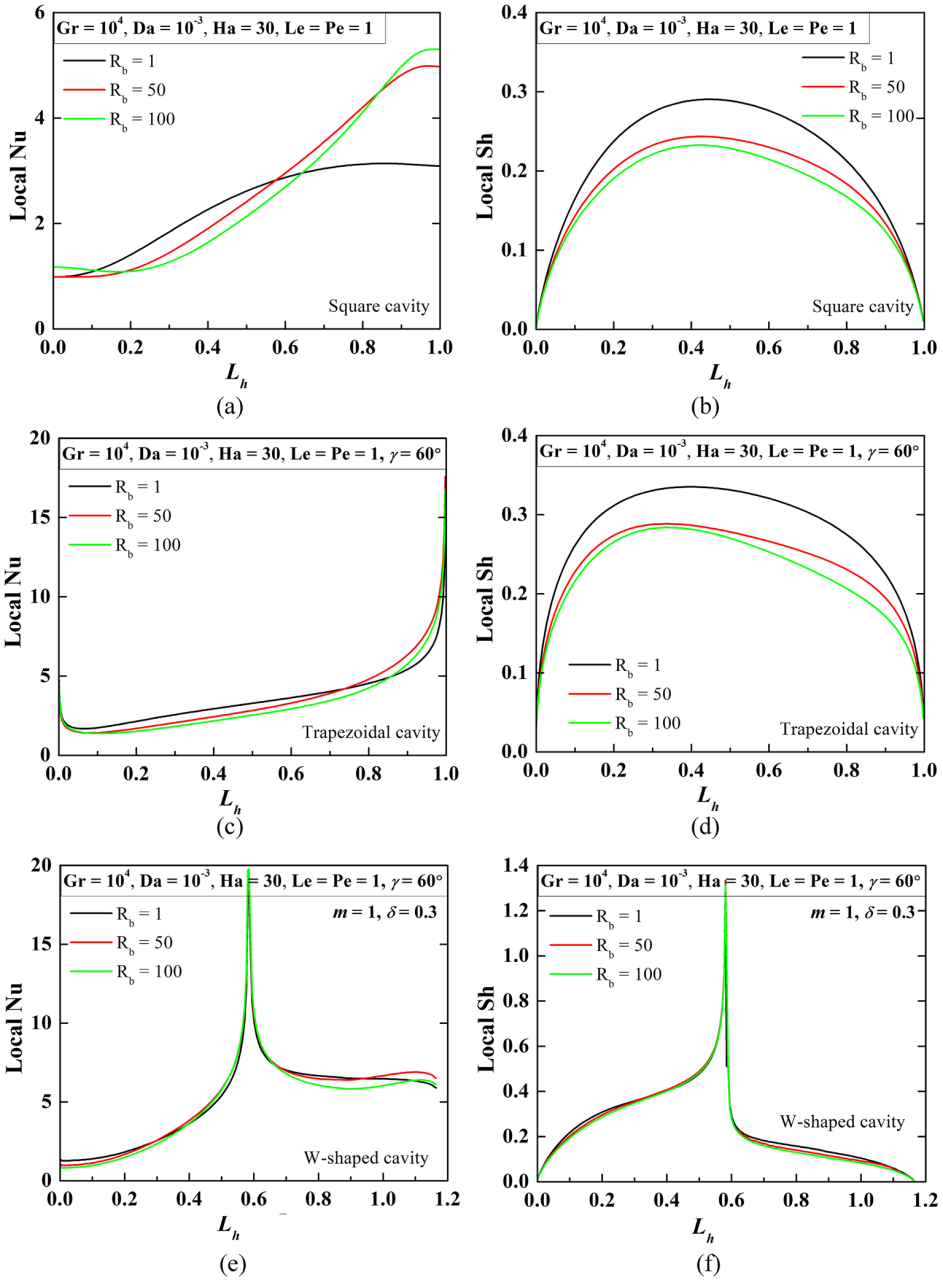
Patterns of local Nu and Sh (over the heated wall) for R_b_ = 1, 50, 100 at Le = Pe = 1, Gr = 10^4^, Da = 10^–3^, Ha = 30 for square (**a**, **b**), trapezoidal (**c**, **d**), and W-shaped (e–f) cavities.

The local Nu as well as Sh values are plotted by the Fig. 7 at R_b_ = 1, 50, 100 at Le = Pe = 1, Gr = 10^4^, Da = 10^–3^, Ha = 30. The Nu value for the square cavity becomes less on the left side, rises gradually, and becomes maximum at the right wall. This phenomenon indicates high heat transfer to fluid at the right side, its heat transfer rises as R_b_ rises. Due to the high cooling surface of the trapezoidal-shaped cavity, the Nu value at both sides rises. The W-shaped cavity shows a peak Nu value in the middle due to the nature of the shape of the bottom wall. The Sh value also rises more in the middle, this value increases with R_b_ for square and trapezoidal cavities. The phenomenon can be substantiated by the oxygen isoconcentrations as in Fig. 4.

**Figure 8:**
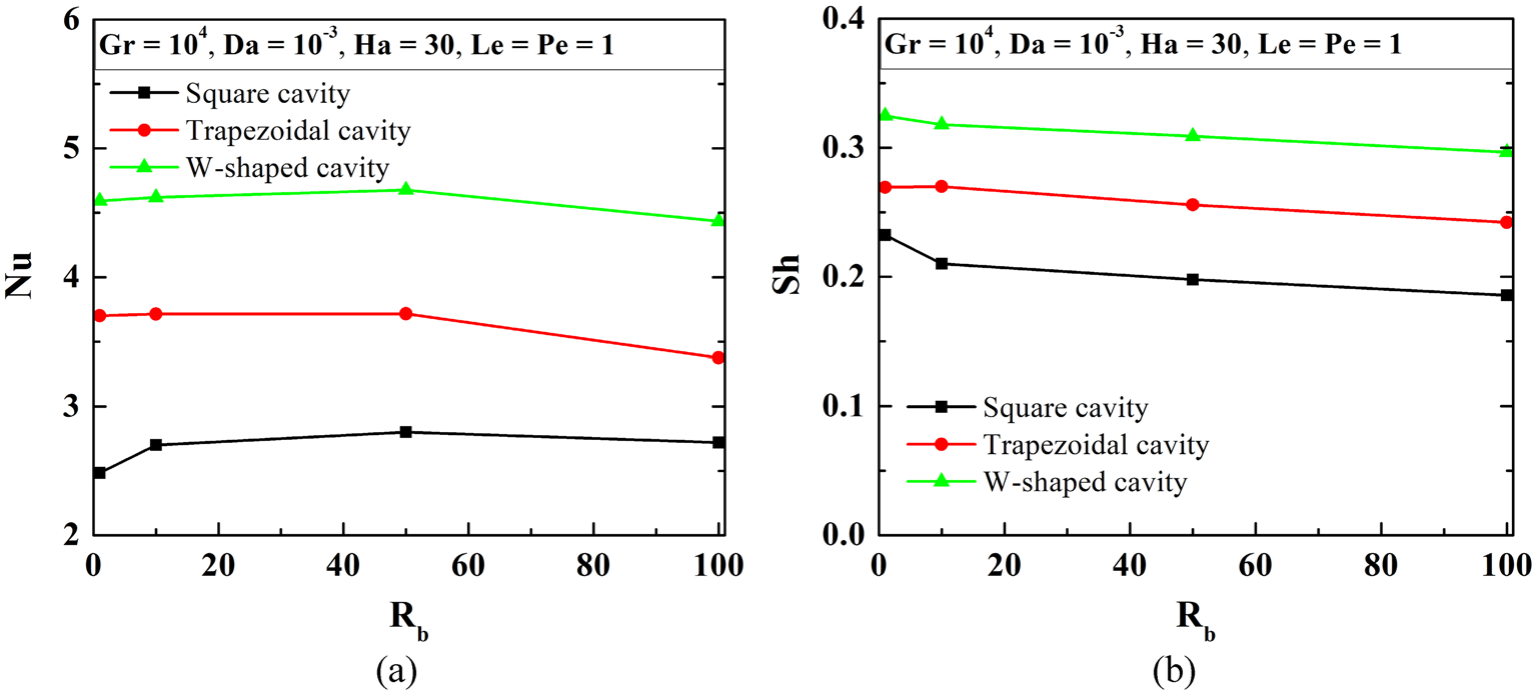
Variation of average values of Nu (**a**), and Sh (**b**) at Le = 1, Pe = 1, Gr = 10^4^, Da = 10^–3^, Ha = 30 changing bioconvection Rayleigh number for square, trapezoidal and W-shaped cavities.

The distribution of average Nu and Sh (as in Fig. 8) reveals a consequent rise in heat transfer and oxygen gradient with a change in shape from a square to a W-shaped cavity. As R_b_ rises, average Nu increases first then drops for all considered shaped cavities. The magnitude of average Sh drops with an increase in R_b_.

### Impact of rise in bottom heating length by *δ*

**Figure 9:**
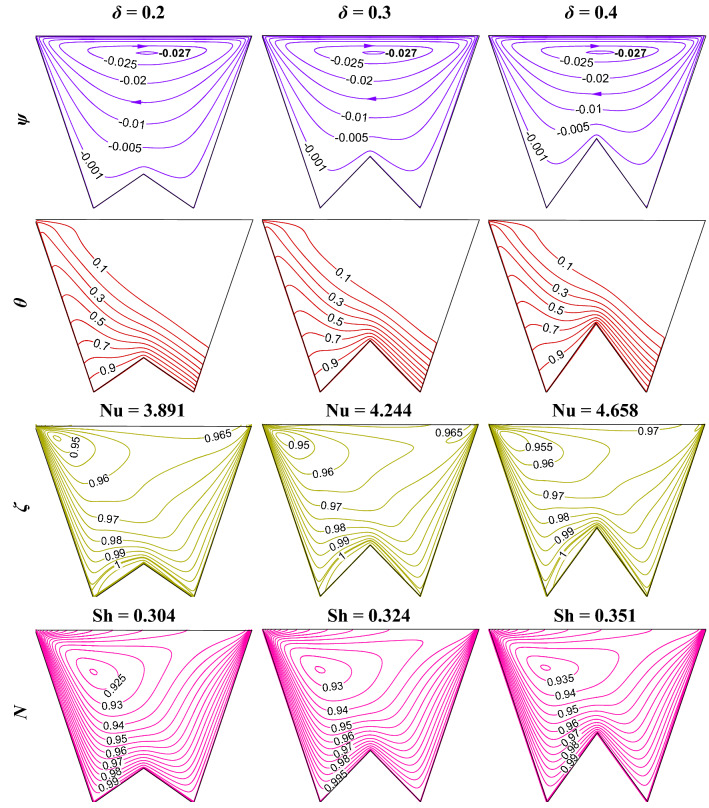
Streamline contours ($\psi$), Isotherms contours ($\theta$), Oxygen isoconcentration ($\zeta$), Microorganisms isoconcentration (*N*) for different triangular bottom heights (*δ*) at R_b_ = 50, Le = Pe = 1, Gr = 10^3^, Da = 10^–3^, Ha = 30, *m* = 1, and $\gamma$ = 60°.

**Figure 10:**
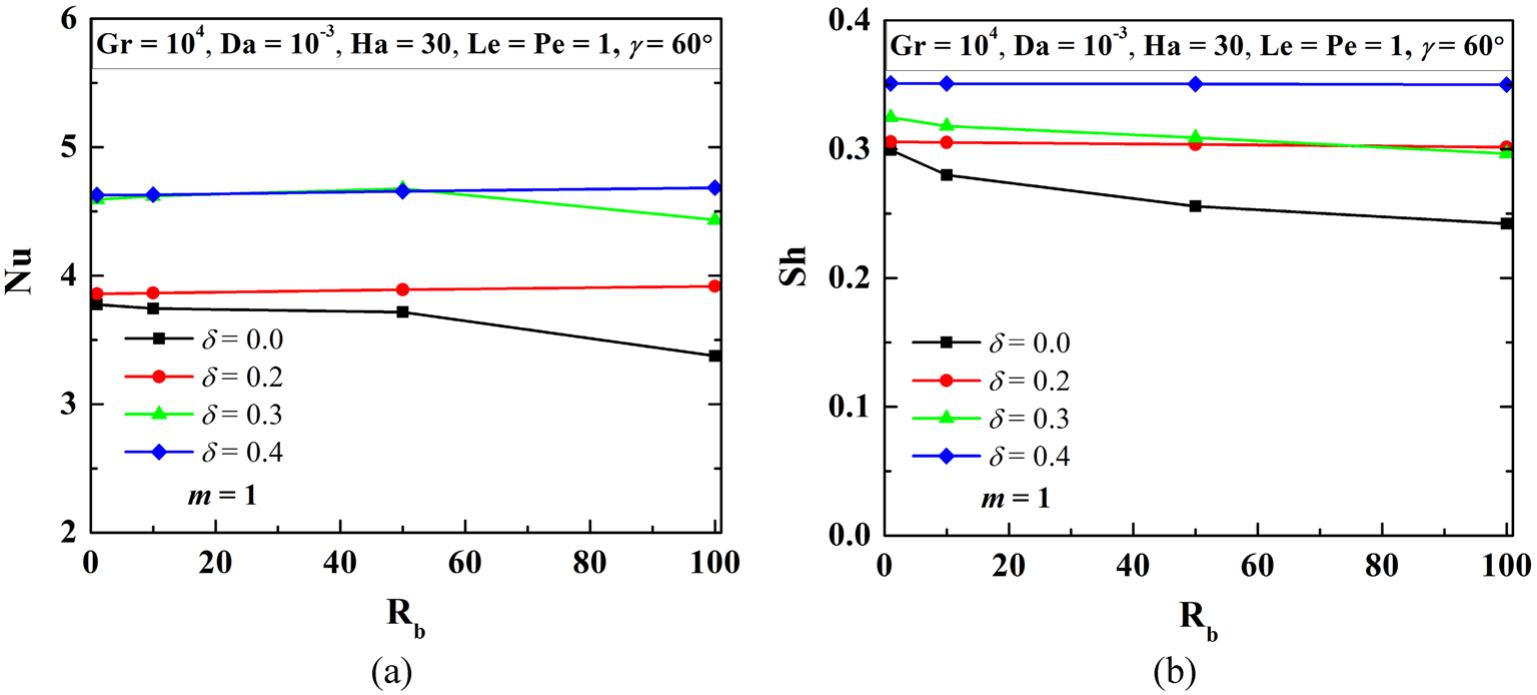
Patterns of average Nu and Sh for different *δ* and R_b_ at Le = Pe = 1, Gr = 10^4^, Da = 10^–3^, Ha = 30, *m* = 1, and $\gamma$ = 60° for W-shaped cavity.

The rise of the bottom-heated surface by *δ* decreases the volume of flowing fluid in the W-shaped enclosure. In fact, with the increase in heating surface, leads to the enhancement in the thermal energy transport that in turn raises the fluid flow strength. Whereas, the reduction in flow volume counteracts the phenomenon. Figure 9 elucidates the impact of *δ* on streamline contours (*ψ*), isotherms contours (*θ*), oxygen isoconcentrations (*ζ*), microorganisms isoconcentrations (*N*) at R_b_ = 50, Le = Pe = 1, Gr = 10^3^, Da = 10^–3^, Ha = 30, *m* = 1 and $\gamma$ = 60°. This study reveals no significant change in streamline strength as *δ* rises. The effect of the increasing area being balanced by the reduction in volume may be the reason for this phenomenon. Isotherms show a higher thermal gradient as *δ* increases, oxygen isoconcentrations move toward the left top corner with an increase in heating surface, and microorganism self-propels accordingly. The magnitudes of average Nu and Sh value rises with *δ* as noted in Fig. 10. As the height of the undulation (bottom triangular shape) increases, heating surface areas also increase, which contributes to the higher heat transfer, as designated by the increasing average Nu (at the lower heated wall), as well as higher oxygen consumption, as indicated by the increasing average Sh (at the lower wall).

### Impact of rise in bottom heating length by undulations (*m*) for W-shaped cavity

**Figure 11:**
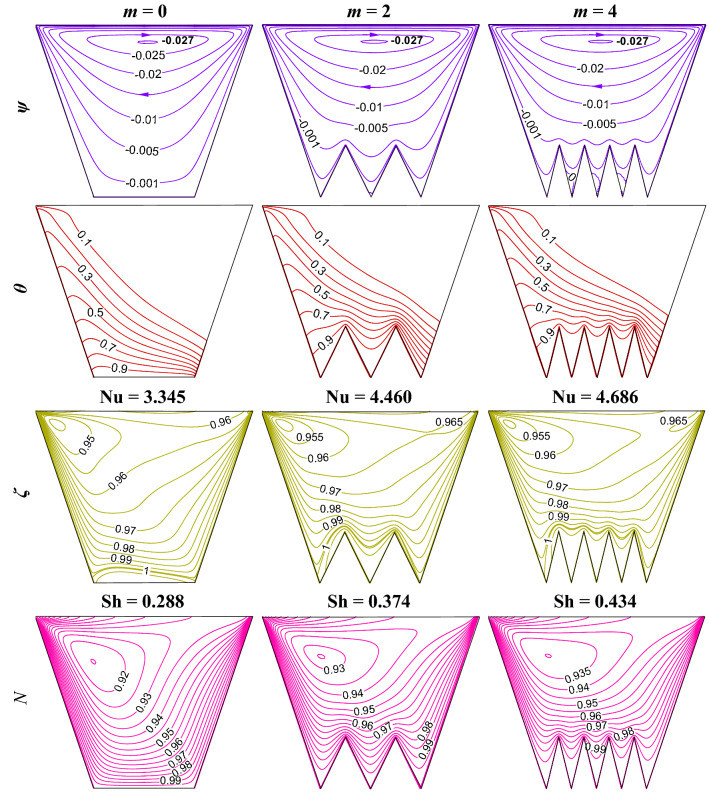
Streamline contours ($\psi$), Isotherms contours ($\theta$), Oxygen isoconcentration ($\zeta$), Microorganisms isoconcentration ($N$) for multiple triangular bottom heights (*n*) at R_b_ = 50, Le = Pe = 1, Gr = 10^3^, Da = 10^–3^, Ha = 30, $\gamma$ = 60°, and *δ* = 0.3.

**Figure 12:**
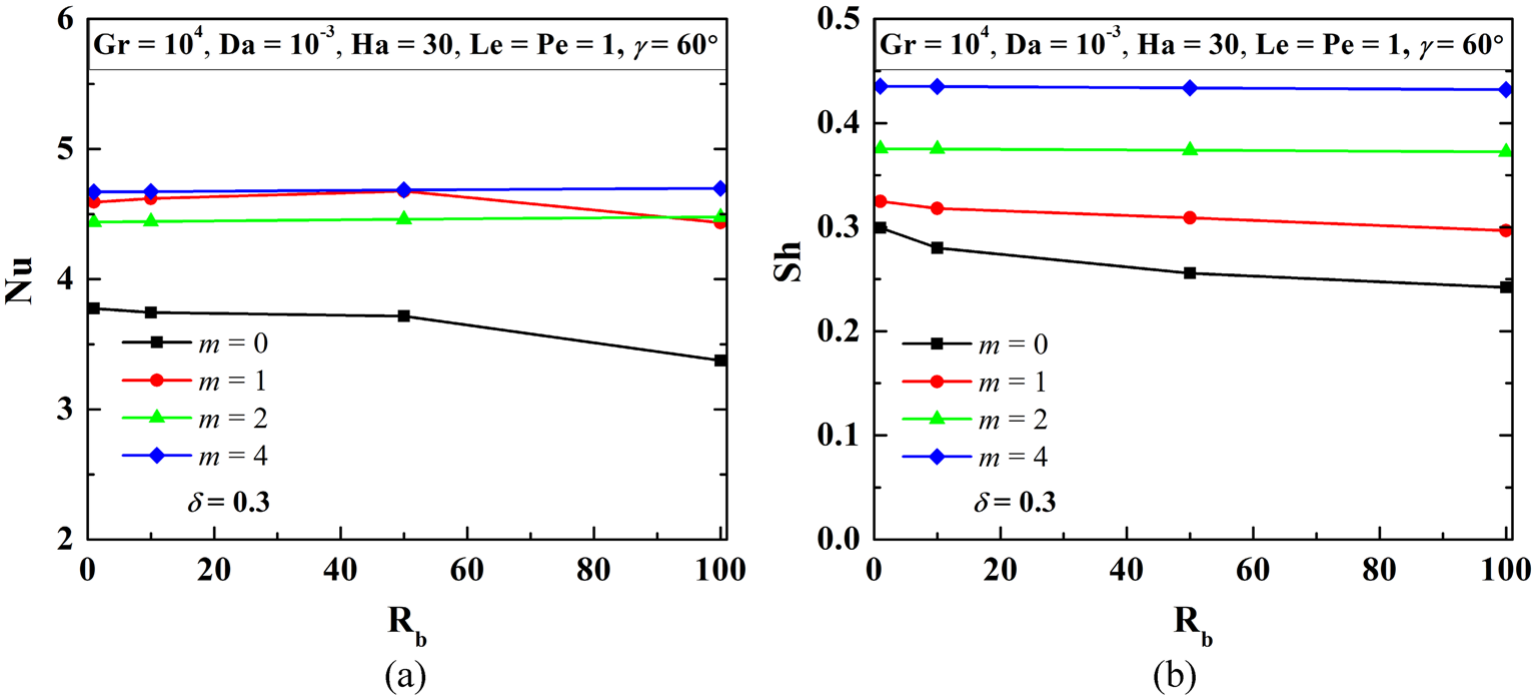
Patterns of average Nu (**a**) and Sh (**b**) for different *m* at Le = 1, Pe = 1, Gr = 10^4^, Da = 10^–3^, Ha = 30, *δ* = 0.3, $\gamma$ = 60^o^ for W-shaped cavity.

The increase in the heating surface area can be achieved by adopting multiple undulations (*m*). For fixed amplitude (*δ* = 0.3) of undulations, fluid flow volume does not change with multiple undulations. However, adding in undulation at the bottom heating wall develops high viscous force with flow separation at the pockets in between the two undulated triangles. The impact of increase in bottom heating length by undulations is illustrated in Fig. 11 using streamline contours ($\psi$), isotherms contours ($\theta$), oxygen isoconcentrations ($\zeta$), microorganisms isoconcentrations (*N*) for multiple triangular bottom heights (*m*) at R_b_ = 50, Le = Pe = 1, Gr = 10^3^, Da = 10^–3^, Ha = 30, $\gamma$ = 60°, *δ* = 0.3. No such variations in the strength of streamlines are noted except for small vortices at the pockets. At the higher undulations numbers, there are tiny flow separations within the pocket in between the two undulations. The temperature gradient is high at the peak of triangular undulation. More undulations lead to the upward movement of oxygen isoconcentrations. This develops a higher gradient at the peak of the bottom triangle. Microorganism’s isoconcentrations self-propels towards the high oxygen zone, this swimming is more as *m* rises. With the increasing number of undulations, streamlines contours, isotherms contours, and isoconcentrations of oxygen and microorganisms become wavy patterns depending near the undulated wall. The variation of average Nu and Sh with the change in *m* has been shown in Fig. 12. It is remarkable to note that the Nu values (average) does not rise always with the rise in *m*. The maximum Nu value is observed at *m* = 1. The decrease in Nu for *m* = 2 and 4 may be due to evolved losses at high undulations. The average Sh increases as undulation rises.

An increase in *δ* or *m* rises heating length (*L*_*h*_), the change in average Nu and Sh by enhancing the heating length, and the corresponding modification of flow volume is summarized in Tables 6 and 7, respectively. All the results are compared with the base case with a square enclosure (*m* = 0, *δ* = 0, $\gamma$ = 90°). In the case of a W-shaped cavity, incorporating *δ* (as in Table 3) enhances the heating area (*L*_*h*_), and lessens the flow volume (*V*_*f*_) for the same cooling length (*L*_*c*_). As a result, the combined effect depicts a rise in Nu and Sh values. The percentage enhancement of Nu for *δ* = 0.2 to 0.3 is higher relative to *δ* = 0.3 to 0.4. For the case of the effect of *m* (as in Table 7), the enhancement of heating length is very large compared to the case of the effect of *δ* (as in Table 6). Working fluid volume remains the same; still, enhancement of heat transfer is not noted too much with multi-undulations. Maximum Nu is revealed at *m* = 1, Nu value drops for a further rise in *m* may be due to huge loss is the reason for this. However, Sh rises as *m* rises.

**Table 6:** Effect of increase in heating length (*L*_*h*_) by *δ* for Le = 1, Pe = 1, Gr = 10^4^, Da = 10^–3^, Ha = 30, $\gamma$ = 60°.

Criteria	*m*	*δ* = 0	*δ* = 0.2	*δ* = 0.3	*δ* = 0.4
Heating length (*L*_*h*_)	1	1	1.077	1.166	1.280
Increase in heating length		0	0.077	0.166	0.280
% of increase in heating length		0	7.700	16.600	28.000
Average Nu		3.716	3.859	4.593	4.628
Average Nu increase		0	0.143	0.877	0.912
% of increase in average Nu		0	3.841	23.600	24.541
Average Sh		0.256	0.306	0.325	0.351
Increase in average Sh		0	0.050	0.069	0.095
% of increase in average Sh		0	19.402	26.871	37.129
Working fluid volume (*V*_*f*_)		1	1.475	1.425	1.375
Increase in fluid volume		0	0.475	0.425	0.375
% of increase in fluid volume		0	47.500	42.500	37.500

**Table 7:** Impact of increase in heating length (*L*_*h*_) by *m* for Le = 1, Pe = 1, Gr = 10^4^, Da = 10^–3^, Ha = 30, *δ* = 0.3, $\gamma$ = 60°.

Criteria	*δ*	*m* = *0*	*m* = 1	*m* = 2	*m* = 4
Heating length (*L*_*h*_)	0.3	1	1.166	1.566	2.600
Increase in heating length		0	0.166	0.566	1.600
% increase in heating length		0	16.600	56.600	160.000
Average Nu		3.716	4.678	4.460	4.386
Average Nu increase		0	0.962	0.744	0.670
% of increase in average Nu		0	25.878	20.023	18.030
Average Sh		0.256	0.309	0.374	0.434
Increase in average Sh		0	0.053	0.118	0.178
% of increase in average Sh		0	20.695	46.043	69.445
Working fluid volume		1	1.425	1.425	1.425
Increase in fluid volume		0	0.425	0.425	0.425
% of increase in fluid volume		0	42.500	42.500	42.500

### Influence of increase in cooling length (*L*_*c*_) by sideangle (*γ*)

**Figure 13:**
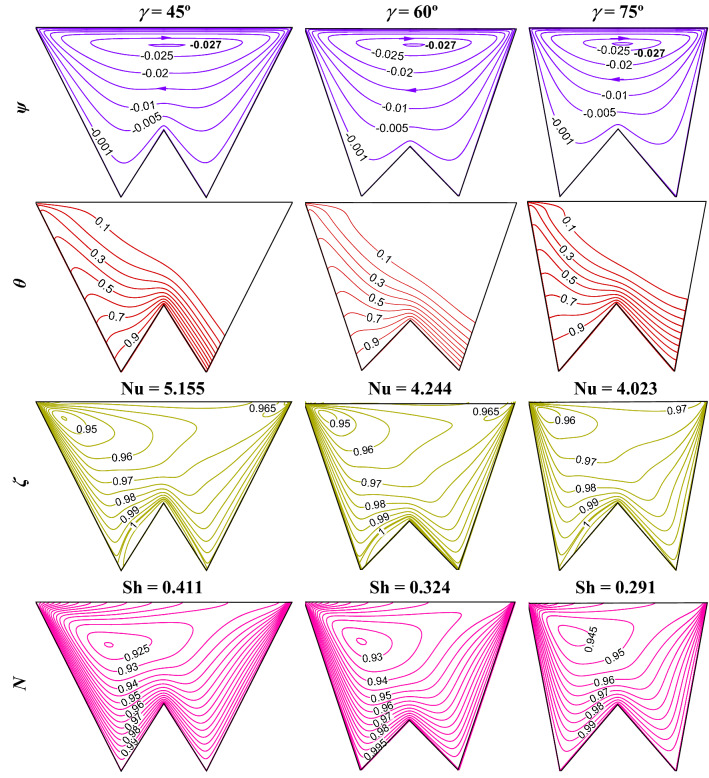
Streamline contours ($\psi$), Isotherms contours ($\theta$), Oxygen isoconcentration ($\zeta$), Microorganisms isoconcentration ($N$) for different side angles ($\gamma$) at R_b_ = 50, Le = Pe = 1, Gr = 10^3^, Da = 10^–3^, Ha = 30, *m* = 1, *δ* = 0.3.

**Figure 14:**
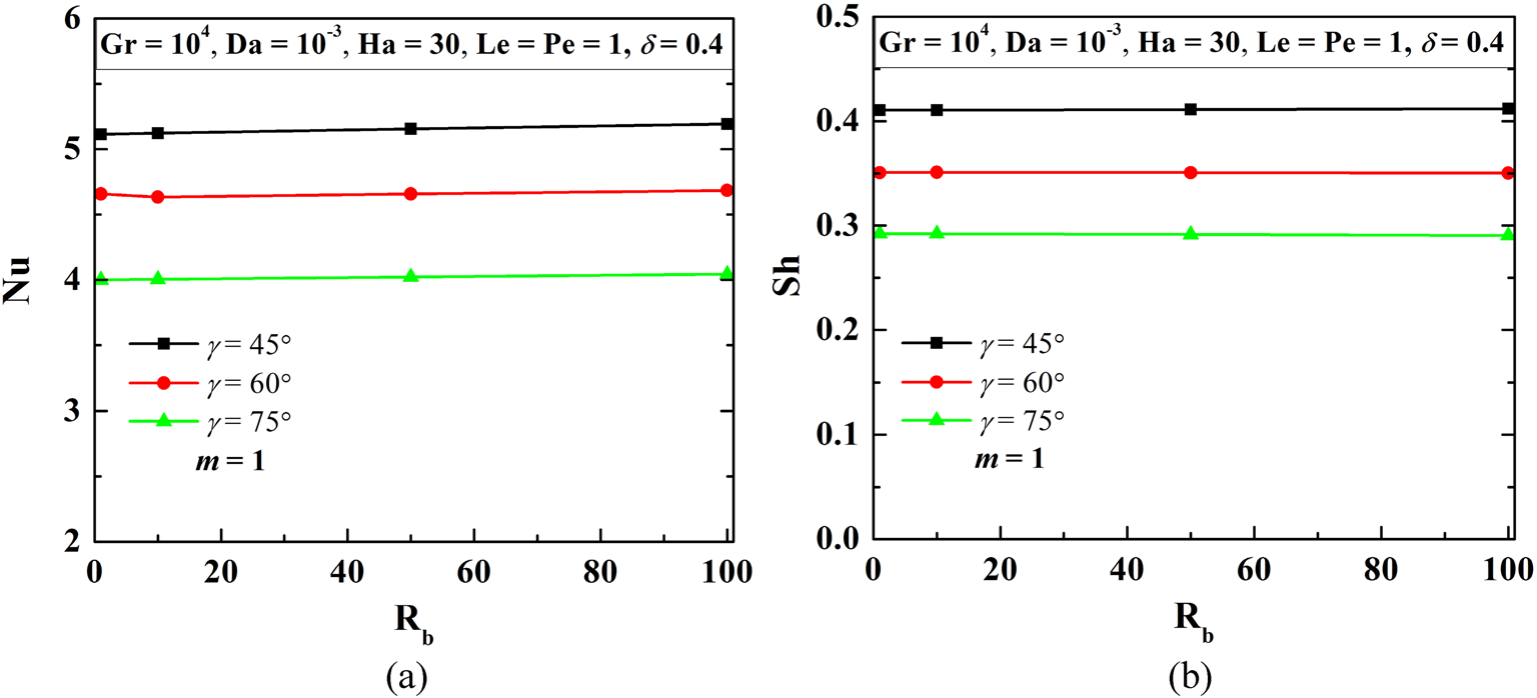
Patterns of average Nu (**a**), and Sh (**b**) when $\gamma$ at Le = 1, Pe = 1, Gr = 10^4^, Da = 10^–3^, Ha = 30, *m* = 1, *δ* = 0.4, for W-shaped cavity.

Since the cooling length (*L*_*c*_) is another parameter for controlling the thermal energy transport and associated mass transfer, therefore, this section focuses on the variation of the top cooling wall by modifying the side angle *γ* of the W-shaped cavity*.* Increase in *γ*, the cooling length as well as working fluid flow volume (*V*_*f*_) drops. The impact of such phenomenon is presented in Fig. 13 using streamline contours ($\psi$), isotherms contours ($\theta$), isoconcentrations of oxygen ($\zeta$) and microorganisms (*N*) at R_b_ = 50, Le = 1, Pe = 1, Gr = 10^3^, Da = 10^–3^, Ha = 30, *m* = 1, *δ* = 0.3. No such impact is noted for the streamlines, however, isotherms show significant changes that correspond to heat transfer and isotherm gradient at wall drops. Because of flow reduction, movement of oxygen isoconcentrations is pronounced, as $\gamma$ rises. The higher concentration area of oxygen leads the microorganism movement towards the left upper corner of the cavity. The average Nu and Sh values drop as $\gamma$ increases are noted in Fig. 14. It is noted from Table 8 that the rate of decrease of cooling length and volume is uniform but the decrement of Nu is not uniform, As $\gamma$ increases, the Sh value decreases uniformly and is uniform.

**Table 8:** Overall assessment with the increasing cooling length (*L*_*c*_) by *γ* when Le = Pe = 1, Da = 10^–3^, Gr = 10^4^, Ha = 30, *m* = 1, δ = 0.3.

Criteria	*m*	$\gamma$ = 0	$\gamma$ = 45°	$\gamma$ = 60°	$\gamma$ = 75°
Cooling length (*L*_*c*_)	1	1	3	2.15	1.53
Cooling length increment		0	2	1.15	0.53
% of increase in cooling length		0	200	115	53
Average nu		3.716	5.155	4.658	4.023
Average nu increment		0	1.439	0.942	0.307
% of increase in average Nu		0	38.734	25.347	8.254
Average Sh		0.256	0.411	0.351	0.291
Average Sh increment		0	0.155	0.095	0.035
% of increase in average Sh		0	160.613	136.973	113.836
Working fluid volume (*V*_*f*_)		1	2	1.57	1.265
Increase in fluid volume		0	1	0.57	0.265
% of increase in fluid volume		0	100	57	26.5

### Impact of change in Darcy (Da) and Hartmann (Ha) numbers

**Figure 15:**
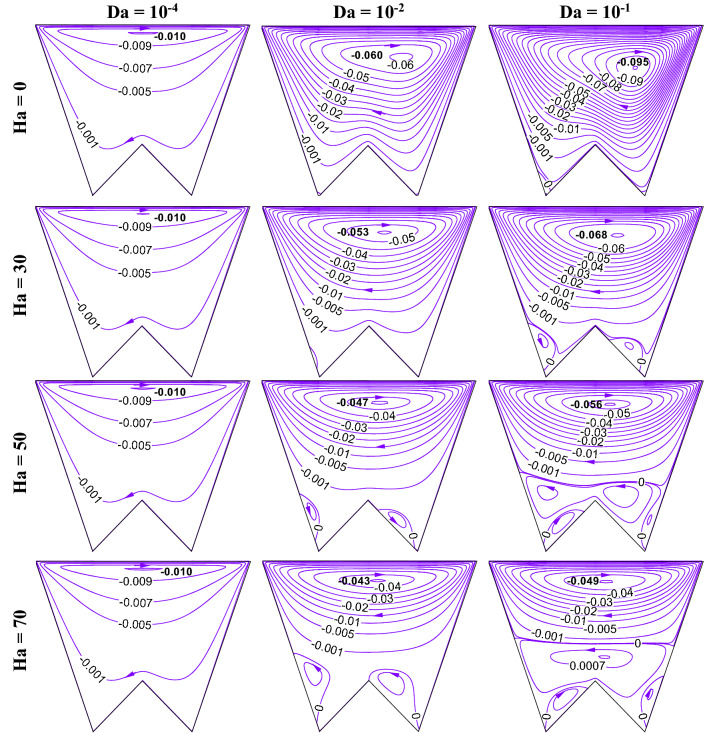
Contours of streamline ($\psi$) at R_b_ = 50, Le = Pe = 1, Gr = 10^3^, *m* = 1,$\lambda$ = 0.3, $\gamma$ = 60°.

**Figure 16:**
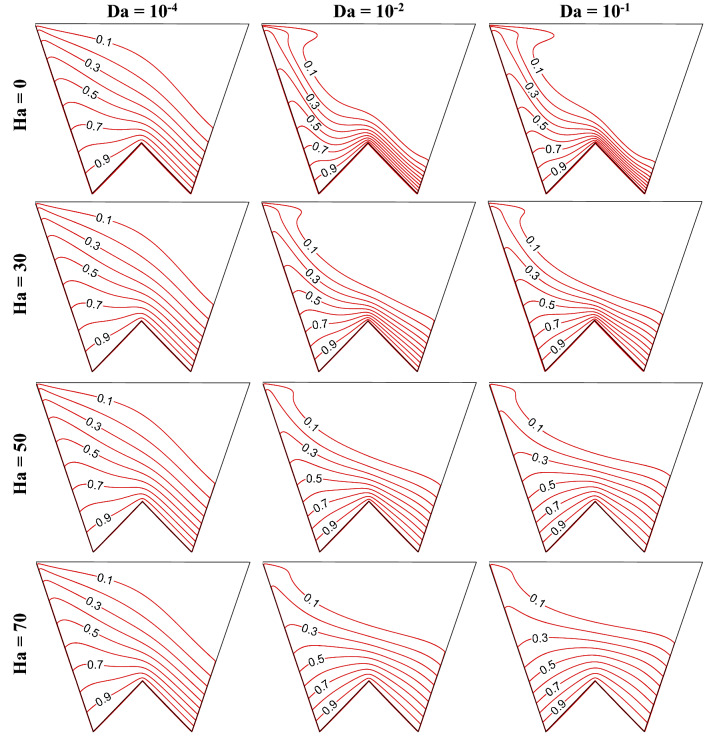
Isotherm contours ($\theta$) at R_b_ = 50, Le = Pe = 1, Gr = 10^4^, *m* = 1, *δ* = 0.3, $\gamma$ = 60°.

**Figure 17:**
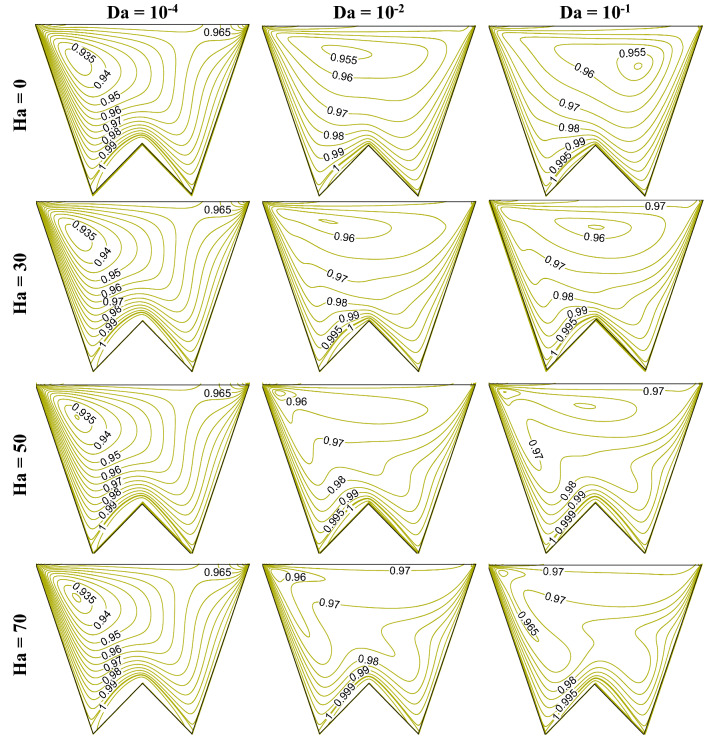
Isoconcentration of oxygen ($\zeta$) at R_b_ = 50, Le = 1, Pe = 1, Gr = 10^4^, *m* = 1, *δ* = 0.3, $\gamma$ = 60°.

**Figure 18:**
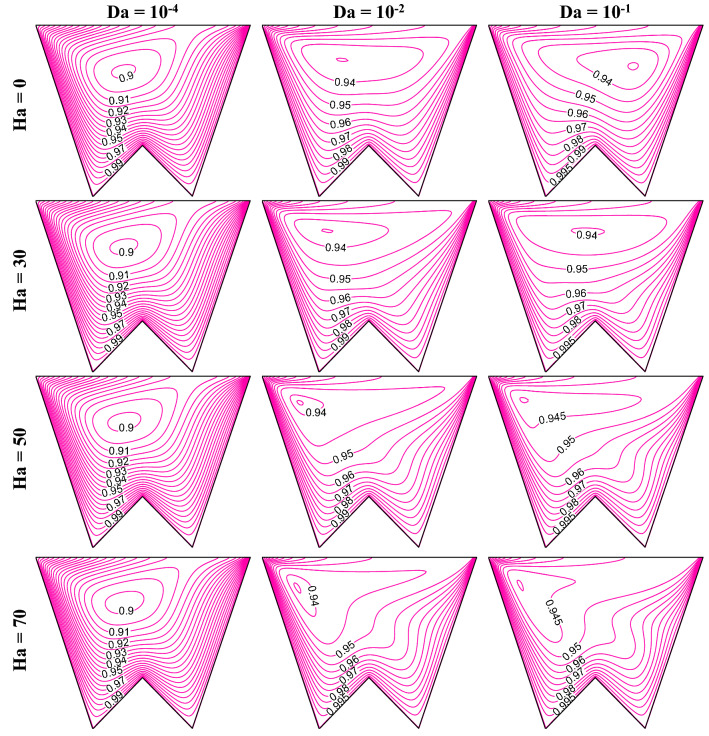
Isoconcentration of microorganisms (*N*) at R_b_ = 50, Le = 1, Pe = 1, Gr = 10^4^, *m* = 1, *δ* = 0.3, $\gamma$ = 60°.

**Figure 19:**
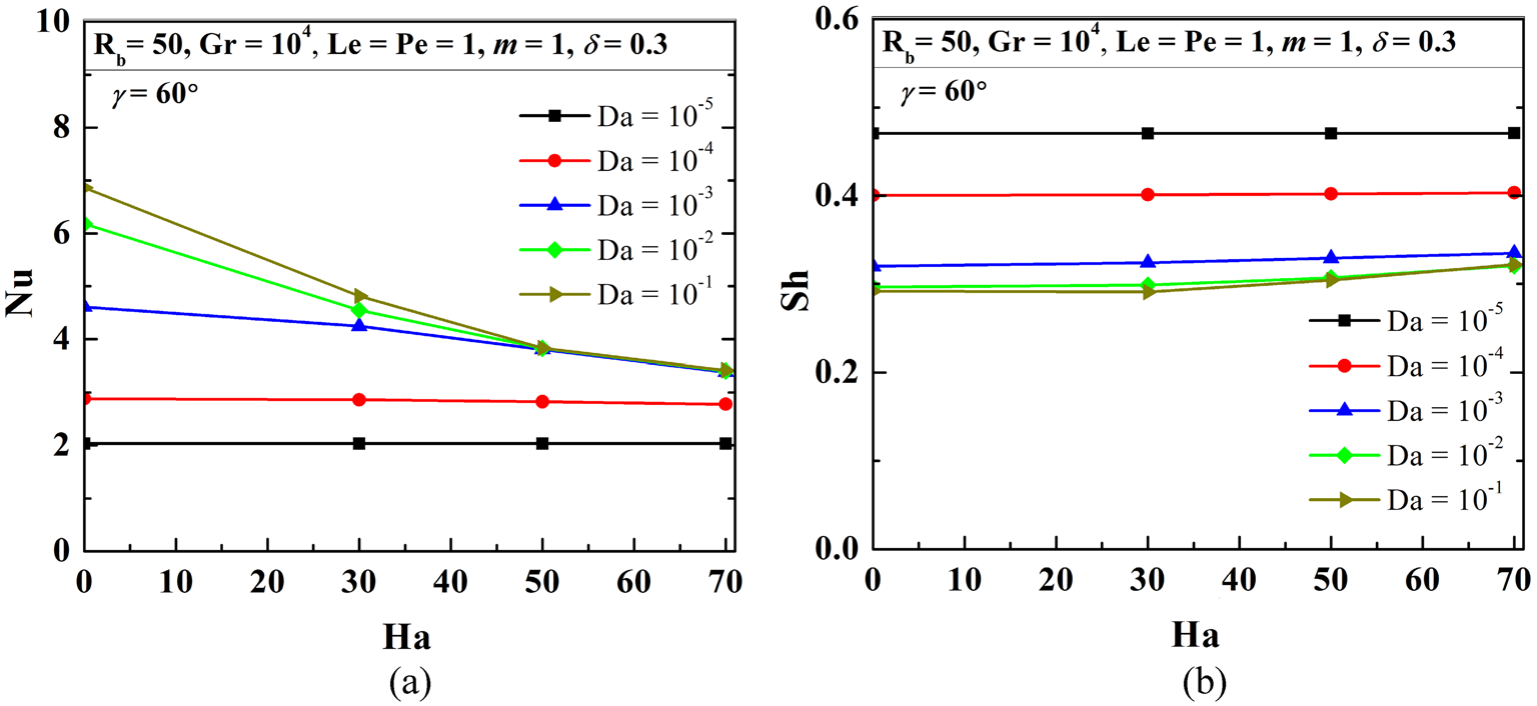
Variations of global Nu (**a**), and Sh (**b**) when R_b_ = 50, Le = Pe = 1, Gr = 10^4^, *m* = 1, *δ* = 0.3, $\gamma$ = 60° changing Hartmann (Ha) and Darcy (Da) numbers.

Increasing Da lessens the flow resistance, Ha weakens the flow velocity, to understand the impact of these numbers, this section presents the streamline contours, isotherms, oxygen and microorganism isoconcentrations at R_b_ = 50, Le = Pe = 1, Gr = 10^3^, *m* = 1, *δ* = 0.3, $\gamma$ = 60° by Figs. 15, 16, 17 and 18, respectively. An increase in flow strength (as in Fig. 15) is pronounced as Da rises by the streamlines for all considered Ha. It can be seen that clockwise circulation strength drops as usual with an increase in Ha. Small vortices (counter-clockwise) are also noted at the bottom of the cavity owing to the net effect of flow separation plus the weakening of flow. At the higher Da values, the sizes of lower circulating cells rise as Ha rises. This is due to the dominating effect of the generated Lorentz force at the higher Ha value even at the lower flow resistance (when Da = 10^–1^). Therefore, triple convective flow dynamics are modulated by the flow controlling parameters also. A significant alteration in isotherms (as in Fig. 16) is noted as Da rises for all considered ranges of Ha. The isotherm lines become more grouped over the bottom heated wall; this rises the low-temperature at the upper right corner. When Ha rises, the thermal boundary thickness rises indicating less heat transfer from the heated wall to the working fluid. An increase in Da value significantly changes the isoconcentrations of oxygen (in Fig. 17), and the vortex shifts from left to rightward that in turn changes oxygen density. This happens due to a rise in flow strength. The rise in Ha value shows a reverse phenomenon, the vortex moves back to the left top corner, due to the weakening of flow. Since the presence of oxygen attracts microorganisms, microorganism isoconcentrations (as in Fig. 18) shows a similar trend of movement of microorganism. The variation of average Nu depicts (in Fig. 19a) the rise in Nu magnitude as Da rises, maximum Nu is noted at low Ha. The Sh value (as in Fig. 19b) drops for the rise in Da, with no significant change (small decrease) with Ha as observed in the Fig. 19.

### Influence of Peclet (Pe) and Lewis (Le) numbers

**Figure 20:**
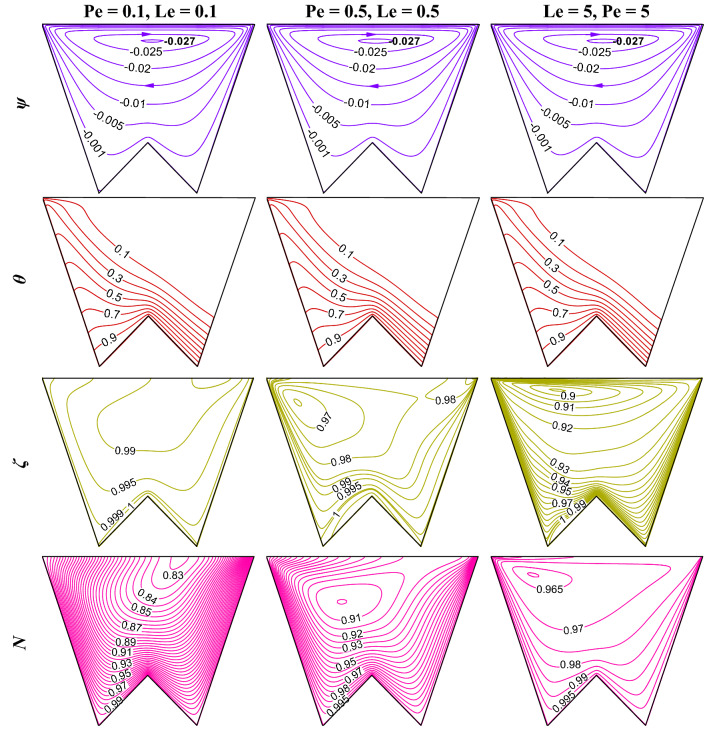
Contours of streamlines (*ψ*), isotherms (*θ*), isoconcentration of oxygen ($\zeta$), and microorganisms ($N$) at R_b_ = 50, Gr = 10^4^, Da = 10^–3^, Ha = 30, *m* = 1, *δ* = 0.3, $\gamma$ = 60°.

**Figure 21:**
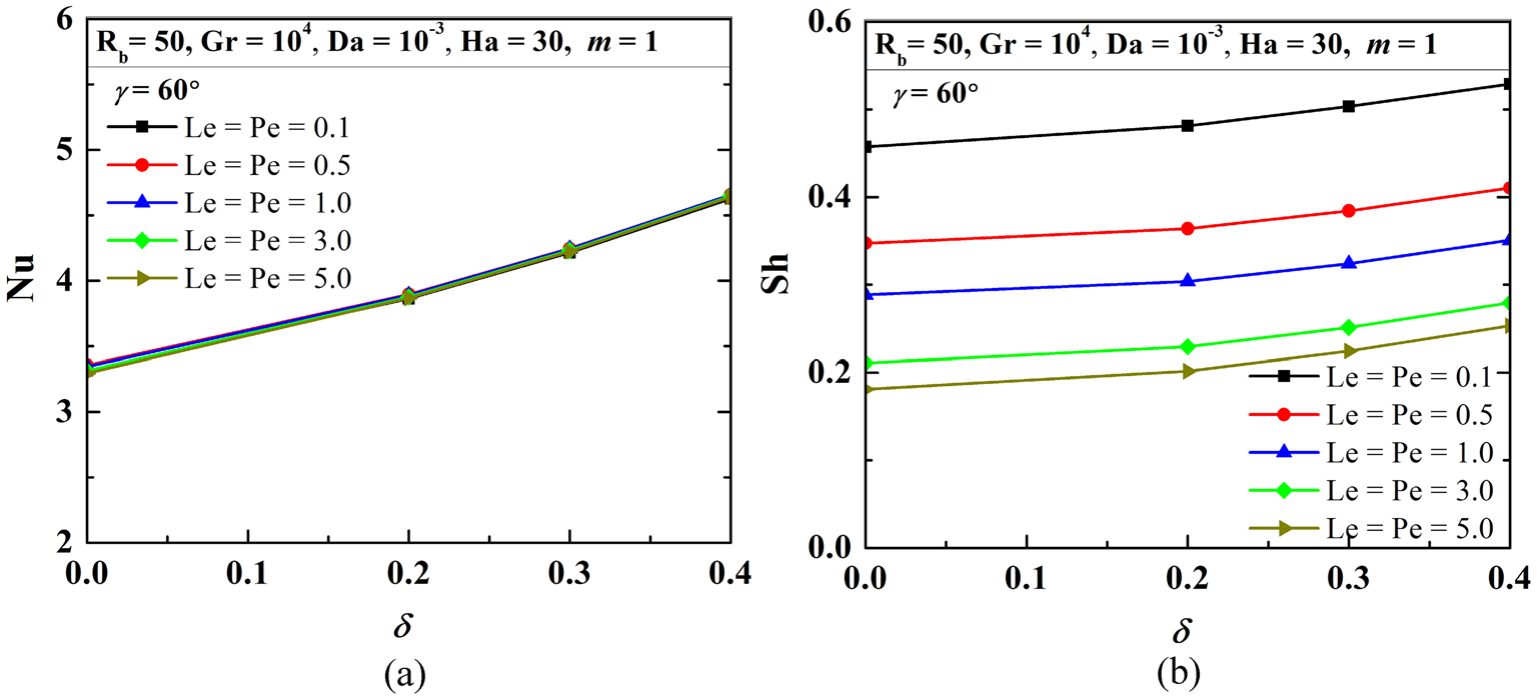
Variations of average Nu (**a**), and Sh (**b**) when R_b_ = 50, Gr = 10^4^, Da = 10^–3^, Ha = 30, *m* = 1, $\gamma$ = 60°, changing Pe and Le.

Since the problem is related to heat and mass transfer, Le and Pe will help to diagnose the flow-physics, with this concept this section presents the flow-structure (*ψ*), temperature (*θ*), isoconcentrations of oxygen (*ζ*) as well as microorganisms (*N*) for the varying Pe = Le = 0.1, 0.5 and 5 when R_b_ = 50, Gr = 10^4^, Da = 10^–3^, Ha = 30, *m* = 1, *δ* = 0.3, $\gamma$ = 60° (as in Fig. 20). No significant difference of streamlines (top-row) and isotherms (second-row) is noted with the increase in Le and Pe. However, for oxygen (third-row) and microorganism (fourth-row) isoconcentrations, different patterns of isoconcentrations are noted for different Le and Pe. An increase in the Pe, as well as Le, isoconcentrations lines, modifies markedly. At the lower Le, Pe variation of the oxygen concentration is less, whereas, at the higher values of Le, Pe variation of the oxygen concentration is more and congested. The isoconcentrations of microorganism follows the existence of oxygen as per the density of oxygen. The magnitude of average Nu (as in Fig. 21a) reveals no substantial variation with Pe and Le, but the increasing trend of average Nu with the increasing *δ* is clearly noted. This is due to the combined effects of the increasing triple convection. Similarly, the value of average Sh drops as Le and Pe rise (as in Fig. 21b), which corresponds to the reduction in oxygen mass transfer at higher Le and Pe. However, average Sh increases as *δ* increases, which is true for any Le and Pe values.

### Influence of Grashof number (Gr)

**Figure 22:**
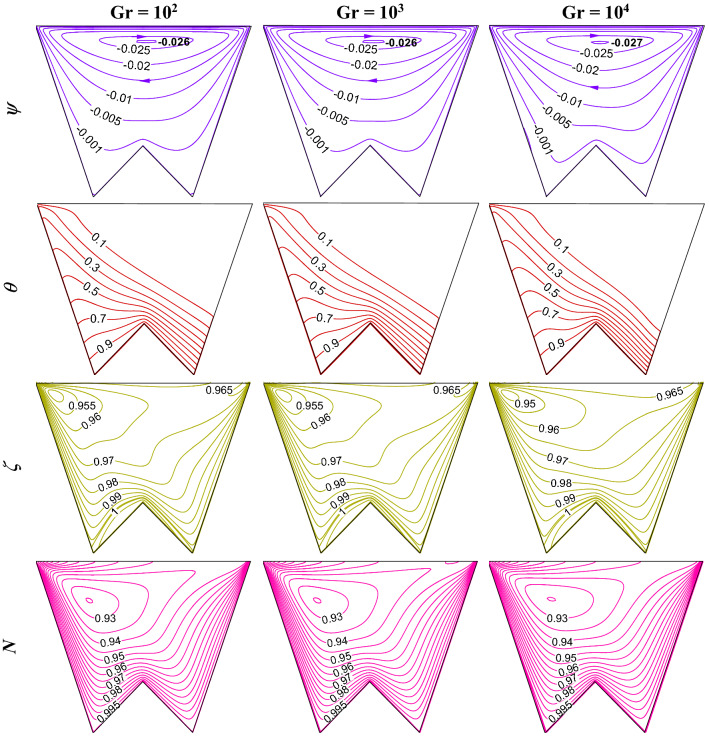
Impact of Grashof number (Gr) on the flow structure (first-row), temperature (second-row), oxygen (third-row), and isoconcentration of microorganisms (fourth-row) when R_b_ = 50, Le = Pe = 1, Da = 10^–3^, Ha = 50, *m* = 1, *δ* = 0.3, $\gamma$ = 60°.

**Figure 23:**
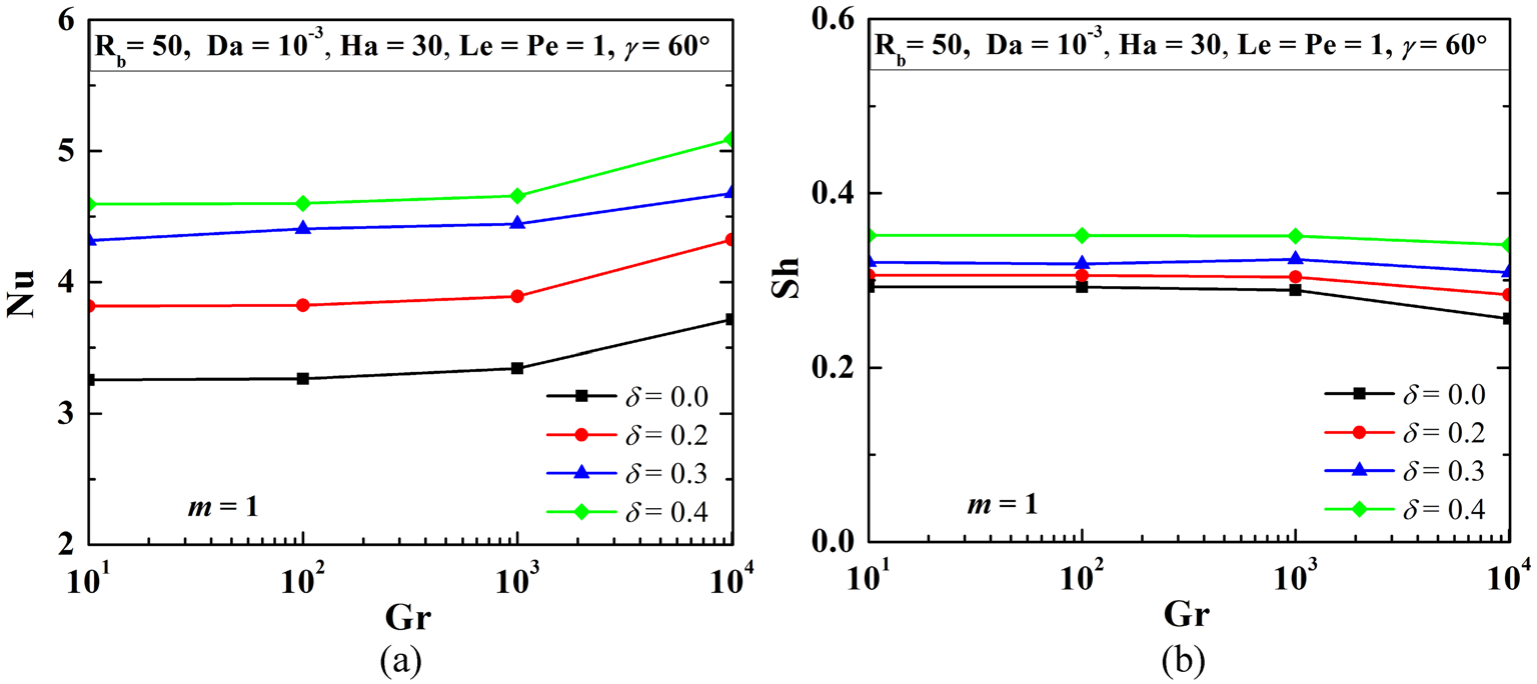
Variations of average Nu (**a**), and Sh (**b**) when R_b_ = 50, Le = Pe = 1, Da = 10^–3^, Ha = 50, *m* = 1, *δ* = 0.3, $\gamma$ = 60° varying Grashof number.

Buoyant convection depicts the strength of the circulating flow field by Gr. Thus the bioconvection study is controlled by Gr and modifies the flow physics accordingly. For the triple convection flow, free convection also modulates the heat and mass transfer. Figure 22 shows the influence of Grashof number (Gr) on the flow structure (first-row), temperature (second-row), isoconcentrations of oxygen ($\zeta$-third-row), and microorganisms (*N*- fourth-row) when R_b_ = 50, Le = Pe = 1, Da = 10^–3^, Ha = 50, *m* = 1, *δ* = 0.3, $\gamma$ = 60°. The rise in Gr enhances the flow strength and shows the crowding of isotherms at the bottom wall. The flow of oxygen diminishes as Gr rises, and microorganism moves accordingly. Figure 23 shows a rise in Nu and a decrease in Sh with the rise in Gr.

A summary of the combined influence of geometric parameters such as number (*m*) and amplitude (*δ*) of the bottom waviness, sidewall inclination (*γ*), and flow controlling parameters such as bioconvection Rayleigh number (R_b_), Grashof number (Gr), Peclet number (Pe), Lewis number (Le), oxygen diffusion ratio ($\chi$), Darcy number (Da), and Hartmann number (Ha) are demonstrated in Table 9. When the undulation amplitude (*δ*) rises when other parameters are fixed, heating length (*L*_*h*_), as well as heat transfer (Nu), increases for the fixed cooling wall length (*L*_*c*_); whereas working fluid volume (*V*_*f*_) and oxygen mass transfer rate (Sh) decreases. However, increase in the undulation numbers (*m*) keeping all other parameters fixed, heating length increases for the fixed cooling wall length and working fluid volume, and both the Nu and Sh increase. However, peak heat transfer is noted for *m* = 1, beyond which heat transfer decreases. The sidewall inclination angle has adverse effects on the triple convective dynamics; the cooling wall length as well as working fluid volume increases but both heat and mass transfer rate decrease. Therefore, it is clearly observed that the geometric parameters (*δ*, *m*, *γ*) have a significant role in the triple convective dynamics in the complex cavity. Now, it is relevant to comment that there is no influence of flow controlling parameters (R_b_, Le, Pe, Da, Ha, and Gr) on the heating/ cooling length, and working fluid volume. For the increasing the bioconvection effect (through R_b_) heat transfer rate increases markedly (keeping all other parameters fixed), while the mass transfer rate declines substantially. On the other hand, Lewis number (Le) and Peclet number (Pe) have a minor impact on the heat transfer, whereas oxygen mass transfer rate reduces with the increasing Le and Pe. As the flow-resistance decreases with the rising Da, heat transfer increases markedly but, oxygen mass transfer rate reduces. Increasing magnetic field strength has adverse effects on both the heat and mass transfer. A rise in the Grashof number causes more thermal convection, that in turn results more heat transfer but less transfer of oxygen.

**Table 9:** Impact of the controlling variables on the overall triple convective behavior of the complex cavity.

Controlling parameters	Heating length (*L*_*h*_)	Cooling length (*L*_*c*_)	Flow volume (*V*_*f*_)	Heat transfer (Nu)	Oxygen transfer (Sh)
_*δ*_	↑	Fixed	↓	↑	↓
_*m*_	↑	Fixed	Fixed	↑ (upto *m* = 1)	↑
$\gamma$	Fixed	↑	↑	↓	↓
R_b_	Fixed	Fixed	Fixed	↑	↓
Le, Pe	Fixed	Fixed	Fixed	No effect	↓
Da	Fixed	Fixed	Fixed	↑	↓
Ha	Fixed	Fixed	Fixed	↓	↓
Gr	Fixed	Fixed	Fixed	↑	↓

## Conclusions

This study numerically explores the triple-convective flow-physics of magnetically susceptible fluid containing copper nanoparticles and oxytactic bacteria in a novel W-shaped porous cavity. The flow physics, heat, and mass transport phenomena are compared with a closer shape like square and trapezoidal-shaped enclosures. Apart from the shape, geometric controlling parameters of the W-shaped cavity (by *m*, *δ*, $\gamma$), the impact of bioconvection Rayleigh number (R_b_), Peclet number (Pe) and Lewis number (Le), porous substance permeability (Da), magnetic field strength (Ha), and Grashof number (Gr) are presented by the flow field ($\psi$), isotherms ($\theta$), oxygen ($\zeta$) and microorganisms (*N)* isoconcentrations. The findings of the study are pointed below:

- This mixed thermo-bioconvection study develops a clockwise circulation, which lies near the top sliding cold wall (due to shear force).
- R_b_ produces secondary anticlockwise circulation near the left adiabatic wall; its size rises as R_b_ increases due to bioconvective phenomena.
- Isotherm lies diagonally from left to right bottom wall with the higher thermal gradient at right wall. This gradient increases with the alteration of the shape of the cavity, square to trapezoidal, trapezoidal to W-shaped cavity.
- Oxygen concentrations tend to move towards the left top corner of the cavity, the propagation is more as R_b_ rises. The concentration of microorganisms follows the presence of oxygen to consume oxygen accordingly.
- The magnitude of heat transfer (average Nu) and oxygen mass transfer (average Sh) for a W-shaped cavity is higher compared to a square and trapezoidal cavity. The average Nu increases (with R_b_) up to a certain value of R_b_, then it drops whereas, Sh drops as R_b_ rises.
- No significant change in the circulation strength is noticed with the variation of the amplitude of the undulation peak (*δ*), however, Nu and Sh value increase as *δ* rises. The influence of *δ* is more compared to a number of undulations (*m*). In general, the shape of a cavity can be considered an important parameter to control the heat and mass transfer process in a novel W-shaped cavity undergoing triple-convective phenomena.
- The average Nu value drops uniformly but the Sh value drops nonuniformly as $\gamma$ increases, which is due to the increased cooling length as well as working fluid volume.
- The impact of porous media permeability (Da) shows high flow strength with a rise in Da, the strength drops as Ha increases (due to the dampening effect of the generated Lorentz force). Significant shifting of oxygen isoconcentrations has been noted as Da rises. At high Da with higher Ha, the flow separation is pronounced at the bottom portion of the cavity.
- The impact of bioconvective Lewis (Le) and Peclet (Pe) numbers on Nu is not too much; however, Sh drops as Le and Pe rise.
- In general, an increase in Gr improves the buoyant convection, which leads to strengthening the fluid flow as well as Nu values, but Sh value drops.

## Data Availability

The data that support the findings of this study are available from the corresponding author upon reasonable request.

## List of symbols

*b*: Chemotaxis parameter, m
*B*: Magnetic field, Wbm^−^^2^
*C*: Oxygen concentration
*C*_*0*_: Oxygen concentration over the side walls
*C*_*min*_: Oxygen concentration required for alive microorganisms (minimum)
*C*_*p*_: Specific heat value
Da: Darcy number
*D*_*c*_: Oxygen diffusivity, m^2^ s^−^^1^
*D*_*n*_: Microorganisms diffusivity, m^2^ s^−^^1^
*F*_*c*_: Forchheimer factor, m^-1^
Gr: Grashof number
*H*: Enclosure height, m
Ha: Hartmann number
*K*: Porous structure permeability
*L*: Cavity length, m
Le: Lewis number
*m*: Undulations number
*n*: Motile microorganisms number density (dimensional)
*n*_*o*_: Reference number density
*N*: Motile microorganisms number density (dimensionless)
Nu: Average Nusselt number
*p*: Dimensional pressure, Pa
*P*: Nondimensional pressure
Pe: Peclet number
Pr: Prandtl number
R_b_: Bioconvective Rayleigh number
Re: Reynolds number
Ri: Richardson number, ${\text{Gr/Re}}^{{2}}$
Sh: Sherwood number (average)
*T*: Temperature, K
*u*, *v*: Velocity components, ms^−^^1^
$\tilde{u},\tilde{v}$: Microorganisms velocities, ms^−^^1^
*U*, *V*: Velocity components (dimensionless)
*U*_*t*_: Speed of the sliding wall, ms^−^^1^
*Wc*: Maximum cell swimming velocity
*x, y*: Cartesian coordinate system, m
*X, Y*: Coordinates (dimensionless)

## Greek symbols

$$\alpha$$: Thermal diffusivity, m^2^ s^−^^1^
$\beta$: Expansion coefficient (volumetric), K^−^^1^
$\omega$: Volume of microorganisms (average)
$$\gamma$$: Sidewall inclination
$\delta$: Amplitude of undulations (dimensionless)
$$\varepsilon$$: Porosity
$\zeta$: Dimensionless oxygen concentration
$$\theta$$: Dimensionless temperature
$\mu$: Fluid dynamic viscosity, (N s) m^−2^
$$\nu$$: Viscosity (kinematic), m^2^ s^−1^
$\rho$: Cell/fluid density, kg/m^3^
$\sigma$: Electrical conductivity, μS cm^−1^
$\sigma_{1}$: Fraction of consumption oxygen to diffusion of oxygen
$\phi$: Nanoparticle volume fraction
$\chi$: Oxygen diffusion ratio (constant)
$\psi$: Stream function (dimensionless)

## Subscripts

*c*, *h*: Cold, hot
